# Analysis of pCl107 a large plasmid carried by an ST25 *Acinetobacter baumannii* strain reveals a complex evolutionary history and links to multiple antibiotic resistance and metabolic pathways

**DOI:** 10.1093/femsmc/xtac027

**Published:** 2022-11-18

**Authors:** Rayane Rafei, Jonathan Koong, Marwan Osman, Ahmad Al Atrouni, Monzer Hamze, Mehrad Hamidian

**Affiliations:** Laboratoire Microbiologie Santé et Environnement (LMSE), Doctoral School of Science & Technology, Faculty of Public Health, Lebanese University, Tripoli 1300, Lebanon; Australian Institute for Microbiology and Infection, University of Technology Sydney, Ultimo NSW 2007, Australia; Cornell Atkinson Center for Sustainability, Cornell University, Ithaca, NY 14853, United States; Department of Public and Ecosystem Health, College of Veterinary Medicine, Cornell University, Ithaca, NY 14853, United States; Laboratoire Microbiologie Santé et Environnement (LMSE), Doctoral School of Science & Technology, Faculty of Public Health, Lebanese University, Tripoli 1300, Lebanon; Laboratoire Microbiologie Santé et Environnement (LMSE), Doctoral School of Science & Technology, Faculty of Public Health, Lebanese University, Tripoli 1300, Lebanon; Australian Institute for Microbiology and Infection, University of Technology Sydney, Ultimo NSW 2007, Australia

**Keywords:** *Acinetobacter baumannii*, antibiotic resistance, ST25, BREX, *ptx*, uric acid

## Abstract

*Acinetobacter baumannii* has successfully spread during the last decades as one of the main critically important pathogens. However, many aspects including plasmids, are still under-investigated. Here, we report the complete sequence of an *Acinetobacter baumannii* strain, belonging to the ST25^IP^ (Institut Pasteur) sequence type recovered in 2012 in Lebanon, using a combination of Illumina MiSeq and Oxford Nanopore sequencing and a hybrid assembly approach. This strain (Cl107) carries a 198 kb plasmid called pCl107 that encodes the MPF_I_ conjugative transfer system. The plasmid carries the *aacA1, aacC2, sul2*, *strAB*, and *tetA*(B) antibiotic resistance genes. pCl107 region encompassing the *sul2*, *strAB*, *tetA*(B) is closely related to AbGRI1 chromosomal resistance islands, which are widespread in *A. baumannii* strains belonging to Global Clone 2. The resistance region found in pCl107 is one of the missing links in the evolutionary history of the AbGRI1 islands. pCl107 also contains a BREX Type 1 region and represents one of the two main evolution patterns observed in BREX clusters found in plasmids related to pCl107. pCl107 also harbours a *ptx* phosphonate metabolism module, which plays an ancestral structure compared to other large plasmids in ST25 strains. While the uric acid metabolic module found in pCl107 is incomplete, we identified possible ancestors from plasmids and chromosomes of *Acinetobacter* spp. Our analyses indicate a complex evolutionary history of plasmids related to pCl107 with many links to multiple antibiotic resistance and metabolic pathways.

## Introduction

*Acinetobacter baumannii* is an important nosocomial pathogen that continues to attract worldwide attention due to its resistance to many antibiotics and its genomic plasticity, enabling it to acquire genetic material (Imperi et al. 2011, Peleg et al. 2012). Globally, most *A. baumannii* infections are due to the spread of a few clones known as global or international clones (GC) (Zarrilli et al. 2013, Hamidian and Nigro 2019, Koong et al. 2021). Besides the well-studied ST1 (GC1) and ST2 (GC2), members of ST25^IP^ (IP: Institut Pasteur MLST scheme) have been reported as an epidemic, endemic and sporadic in many regions around the world (Sahl et al. 2015, Hamidian and Hall 2016, Sennati et al. 2016, Nigro and Hall 2017, da Silva et al. 2018, Hamidian and Nigro 2019). ST25 was first described as a singleton (a sequence type without known single-locus variants (SLV)) (Karah et al. 2012), but few SLV, e.g. ST402 and ST991, have then been reported hinting for further diversification of this well-established sequence type (Cerezales et al. 2020). In addition to their epidemiological importance, ST25 strains have gained special attention mainly due to increasing antimicrobial resistance, particularly to carbapenems (Zarrilli et al. 2013, Sahl et al. 2015, Cerezales et al. 2020), as well as their ability to produce extensive biofilm compared to other globally distributed clones (Sahl et al. 2015).

Plasmids can significantly impact the biology of *A. baumannii* by introducing additional genes encoding antibiotic resistance and metabolic functions (Fondi et al. 2010). Two plasmids (pD4 and pD46-4; GenBank acc. no. KT779035 and MF399199, respectively) characterized in two Australian ST25 strains are amongst those that carry a large number of genes involved in various functions (Hamidian and Hall 2016, Nigro and Hall 2017). pD4 and pD46-4 belong to a broad and diverse family of conjugative plasmids that encode the MPF_I_ conjugative transfer system and several important functions involved in many cellular pathways, e.g. Type 6 Secretion System (T6SS) (Weber et al.^2015^, Hamidian and Hall 2016, Nigro and Hall 2017).

Understanding the biology and evolution of *A. baumannii* is vital in developing therapeutic agents and preventing infections. However, knowledge of *A. baumannii* strains is restricted due to the biased study of its different biological aspects. Genomic studies often look for antimicrobial resistance mechanisms and ignore other potentially novel gene clusters that remain buried within the genomic data, including metabolic genes. Over the last decade, genome sequencing has superseded traditional methods to track and characterize microbial pathogens. However, while *A. baumannii* genomes are being sequenced swiftly in many countries; the genomic data are still scarce from many regions, such as the Middle East (Hamidian and Nigro 2019, Koong et al. 2021). Wars have served, not only at the Middle East but at the worldwide level, as a breeding ground (Rafei et al. 2014, 2015) for many pathogens as *A. baumannii* that take advantage of the weak health infrastructure and shattered infection control measures (Rafei et al. 2014, Ismail et al. 2018, Higgins et al. 2020). *A. baumannii* strains recovered in the Middle East region are important given that this region has been suggested as a potential source for the spread of some of the globally distributed GC1 lineages (Griffith et al. 2007, Calhoun, Murray and Manring 2008, Koong et al. 2021).

In Lebanon, ST25 was one of the commonly identified sequence types in clinical settings, although at a lesser degree than the dominant sequence types (e.g. ST2 strains) (Rafei et al. 2015, Al Atrouni et al. 2016, Chamieh et al. 2019). Currently, no complete Middle Eastern ST25 genomes are available in GenBank (Table S1). Hence, we completed and analyzed the genome sequences of one ST25 isolate from Lebanon, strain Cl107. We showed that it contains a large 198 kb plasmid with several important modules, including two antibiotic resistance clusters, a *ptx* operon (involved in phosphate metabolism), a BREX (phage resistance) system, and a uric acid metabolic operon. These regions were analyzed in detail. We also explored publicly available complete plasmids to study related plasmids.

## Material and methods

### Bacterial strain and publicly available genome sequence data used in this study

*A. baumannii* CMUL Cl107 was recovered in 2012 from the urine of an 80-year-old male patient suffering from amyloidosis and anaemia in Tripoli, North Lebanon. Cl107 was previously reported as part of a study investigating the epidemiology of *A. baumannii* strains isolated from different hospitals in Lebanon (Rafei et al. 2015).

### Antibiotic susceptibility and resistance testing

Antibiotic susceptibility was tested by the disc diffusion method according to the recommendations of the Clinical and Laboratory Standards Institute (CLSI) (https://clsi.org/). The tested antibiotics are imipenem (10 µg), ampicillin (25 µg), cefotaxime (30 µg), meropenem (10 µg), ceftazidime (30 µg), ampicillin/sulbactum (10/10 µg), tobramycin (10 µg), spectinomycin (25 µg), gentamicin (10 µg), netilmicin (30 µg), neomycin (30 µg), kanamycin (30 µg), streptomycin (25 µg), amikacin (30 µg), sulphamethoxazole (100 µg), rifampicin (30 µg), trimethoprim (5 µg), ceftriaxone (30 µg), nalidixic acid (30 µg), ciprofloxacin (5 µg), florfenicol (30 µg), chloramphenicol (30 µg), and tetracycline (30 µg). Resistance to colistin was measured using ETEST® (https://www.biomerieux.com.au/product/etestr) according to the manufacturer's instructions and interpreted according to CLSI breakpoints. Resistance profiles were interpreted according to the CLSI guidelines for *Acinetobacter* spp. and CDS disk diffusion assay (http://cdstest.net) when a CLSI breakpoint for *Acinetobacter* spp. was not available (e.g. for neomycin, netilmicin, streptomycin, spectinomycin, kanamycin, trimethoprim, sulfamethoxazole, nalidixic acid, florfenicol, chloramphenicol, and rifampicin).

### Whole-genome sequencing, assembly, and annotation

The DNA extraction was done with QIAGEN DNeasy genomic DNA extraction Kit following the manufacturer's instructions. Short-read library preparation was done using the Nextera (DNA Flex library preparation) library preparation kit and sequenced using Illumina MiSeq. MinION (Oxford Nanopore) long-read sequencing was done following library preparation using the Rapid Barcoding Sequencing Kit (SQK-RBK004) and sequenced on an FLO-MIN106D Flow cell according to the manufacturer's instructions (Oxford Nanopore Technologies, Inc., Oxford, UK). Illumina MiSeq generated 1102451 paired-end short reads with 50-fold coverage, an average length of 250 bp, and MinION produced a total of 52680 long reads with an *N_50_* length of 15.2 kbp and 25-fold coverage. FastQC (v.0.11.9) (https://bioinformatics.babraham.ac.uk/projects/fastqc/) and Filtlong (v.0.2.0) (https://github.com/rrwick/Filtlong) were used to check the quality of Illumina and MinION reads, respectively. Filtlong was used to filter long reads with poor quality and those with <1 Kbp length. Reads were trimmed using the Porechop (v.0.2.3) programs using default parameters. The high-quality Illumina and MinION reads were assembled de novo using a hybrid assembly approach using the Unicycler program (v0.4.7) as previously described (Wick et al. 2017).

Protein coding, rRNA, and tRNA gene sequences were annotated using Prokka (Seemann 2014) and RAST (Aziz et al. 2008) on Galaxy (https://usegalaxy.org.au/) and PATRIC (https://www.patricbrc.org/) platforms respectively followed by manual annotations of resistance genes, insertion sequences, CRISPR locus and surface polysaccharide loci using ResFinder (https://cge.cbs.dtu.dk/services/ResFinder/), ISFinder (https://www-is.biotoul.fr/), BLAST (https://blast.ncbi.nlm.nih.gov/), InterPro (https://www.ebi.ac.uk/interpro/search/sequence/), CRISPRCASFINDER (https://crisprcas.i2bc.paris-saclay.fr/CrisprCasFinder/Index) and Kaptive (Wyres et al. 2020) searches. MLST in the Institut Pasteur and Oxford schemes (http://pubmlst.org/abaumannii/) were determined from the genome sequence data. The *bla*_AmpC_ allele was determined using the *Acinetobacter bla*_AmpC_ database available at https://pubmlst.org/. The class B cytochrome P450 was typed using the Cytochrome P450 database available at https://drnelson.uthsc.edu/bacteria/. Figures were drawn to scale using SnapGene^®^ (v6.0.3) and Illustrator (v26.2.1).

### Phylogenetic analysis of the BREX operon

The entire DNA sequences of the *brxA*-*brxL* regions were extracted from genomes that showed homology with that of pCl107. Partial regions with incomplete or frameshifted BREX genes were dismissed from the phylogenetic analysis. In total, 90 entries were retained and aligned using the mafft (v7.457) tool (Katoh and Standley 2013). The length of the BREX operon varied in 90 sequences ranging from 13168 bp to 18839 bp with a mean of 14632 bp and a median of 14562 bp. FastTree (v2.1.11) (Price, Dehal, and Arkin 2009) was used to infer a maximum likelihood phylogenetic tree with GTR+CAT as models. FigTree software (v1.4.3) was used to visualize the final phylogenetic tree (http://tree.bio.ed.ac.uk/software/figtree/).

## Results and discussion

### Antibiotic resistance profile

Cl107 was found resistant to ampicillin, third generation cephalosporins (cefotaxime, ceftazidime, and ceftriaxone), gentamicin, amikacin, tobramycin, tetracycline, nalidixic acid, ciprofloxacin, florfenicol, chloramphenicol, trimethoprim, and sulfamethoxazole (Table S2). Cl107 is susceptible to colistin (with a MIC of <0.25 µg/ml) and ampicillin-sulbactam, imipenem, and meropenem.

### Complete genome sequence of Cl107

The genome sequence of Cl107 was completed using a combination of Illumina MiSeq and Oxford Nanopore (MinION) data following a hybrid assembly approach. The genome assembly consists of a 4056235 bp chromosome and a 198716 bp plasmid named pCl107.

The Cl107 genome belongs to ST229^OX^ (Oxford scheme) and ST25^IP^ (Institut Pasteur scheme). It carries the KL 14 and OCL 6 capsular polysaccharide and lipooligosaccharide outer core, respectively. The genome contains a CRISPR locus belonging to CAS-TypeIFb (Cameranesi, Kurth and Repizo 2022) composed of 6 genes in the CAS cluster (bases 1008607–1017088 of GenBank acc. no. CP098521) and 48 spacers (bases 1017221–1020131 of GenBank acc. no. CP098521). Analysis of the spacers compared to the pool of spacers previously identified and catalogued by Karah *et al*. in 2015 (Karah et al. 2015) revealed that Cl107 contains the CST24 CRISPR locus, which is very similar to CST23, except it lacks any duplicated spacer. According to Karah *et al*. (Karah et al. 2015), CST23-24 constitutes the main subclone of clonal complex 25 with a remarkable ability to be endemic, which has caused national outbreaks in the USA, UAE, Argentina, and Sweden (Karah et al. 2015). Cl107 has the same standard ST25 variants of the intrinsic *bla*_OXA-Ab_ (encoding *bla*_OXA-64_) and *bla*_AmpC_ (99% DNA identity to *bla*_AmpC23_), which are not preceded by an ISAba1 copy. Cl107 was originally reported to carry *bla*_NDM-1_; however, this gene appears to have been lost in its complete genome, likely during the storage or transport to Australia, explaining thus its susceptibility to imipenem (Rafei et al. 2015).

Quinolone resistance accounted for the mutations we found in the *gyrA* DNA gyrase (GyrA S81L) and *parC* topoisomerase IV genes (ParC S84L). These mutations are known to be associated with fluoroquinolones resistance in Gram-negative bacteria, including *A. baumannii* (Vila et al. 1995, 1997, Pérez-Varela et al. 2018).

Several biofilm-related virulence determinants were identified in Cl107, including two *pga* loci (*pgaABCD* and *pgaBCD*), a *csuA/BABCDE* locus (chaperone-usher pili assembly system) regulated by *bfmRS*, an *abaI* gene encoding an autoinducer synthase part of the quorum-sensing system, *ompA*, a *pglL*, a *blp2*, a 5709 bp sized gene encoding BapA prefix-like domain-containing protein, as well as *bap* gene. The *bap* gene of 9315 bp encodes a BAP protein sharing 100% identity with the COOH extremity of type 3 (1169 aa), which is mainly observed in ST25 and differed from type 1 and 2 occurring in GC 1 and 2, respectively, according to De Gregorio *et al*. (De Gregorio et al. 2015).

Cl107 also carries a large plasmid (198716 bp) called pCl107 with a GC% content of 39.13% and 210 coding reading frames. pCl107 encodes several important modules, including the HigB/A toxin/anti-toxin system, the ParA/B and ParM/StbA partitioning protein systems, a DNA primase, a DNA helicase, a DNA topoisomerase, the UmuCD DNA damage repair system, and the BREX-1 phage resistance system. Similar to previously characterized plasmids, such as pD4 and pD46-4 (GenBank acc. no. KT779035 and MF399199, respectively), pCl107 contains several genes associated with the conjugative transfer, including *traIJYWH*, *trbA*, and *trbN* with 100% DNA identity making pCl107 a potentially conjugative plasmid. Sequence analysis showed that pCl107 is related to pA297-3, pD4, pD46-4, and pAB3 (GenBank acc. no. KU744946, KT779035, MF399199, and CP012005, respectively) (Hamidian and Hall 2016, Hamidian, Ambrose and Hall 2016, 2017, Nigro and Hall 2017). The complete sequences of 34 plasmids related to pA297-3, pD4, pD46-4, and pAB3 are available in GenBank non-redundant database (Table 1). Our analysis showed that pCl107 is closely related to pMC1.1 (GenBank acc. no. MK531536), which is a 184 kb plasmid recovered from an ST991 (an SLV of ST25^IP^) isolate from a catheter sample in Bolivia in 2015 (Cerezales et al. 2020). pCl107 carries four genes responsible for aminoglycoside resistance (*strAB*, *aacA1, aacC2*), *sul2* for sulfamethoxazole resistance, and *tetA*(B) gene for tetracycline resistance. pCl107 also carries multiple complete insertion sequences, including three copies of each of ISAba1, ISAba34 and IS*1008*, two copies of each of IS*1007* and ISAba12 and a single copy of each of ISAcsp1, ISAba11, ISAha2, IS*17*, and the common region CR2, along with many truncated insertion sequences, e.g. IS*1006*, ISAba22 and the common region elements CR1, and CR2.

**Table 1.** Distribution of antimicrobial resistance genes, BREX, and some metabolic modules in plasmids related to pCl107.

Plasmid name	Strain name	Size (kb)	ST^IP^	Year	Country	Source	AMR genes	*ptxABCDE* ^a^	*merDACPTR* ^a^	BREX (*brxABC, pglXZ, brxL*)^a^	uric acid cluster^a^	*CYP* ^b^	GenBank acc. no.
p1KSK1	KSK1	218	622	2020	India	Respiratory specimen	*mph-msr(E), armA, strAB, sul1*, *sul2, bla*_PER-7_*, cmlA5, arr-2*	-	-	*ABCX-DUF262-ZL* ^c^	-	+	CP072123
p1KSK2	KSK2	218	622	2020	India	Respiratory specimen	*mph-msr(E), armA, strAB, sul1*, *sul2, bla*_PER-7_,*cmlA5, arr-2*	-	-	*ABCX-DUF262-ZL*	-	+	CP072399
p1KSK6	KSK6	218	622	2020	India	Respiratory specimen	*mph-msr(E), armA, strAB, sul1*, *sul2, bla*_PER-7_*, cmlA5, arr-2*	-	-	*ABCX-DUF262-ZL*	-	+	CP072271
p1KSK7	KSK7	218	622	2020	India	Respiratory specimen	*mph-msr(E), armA, strAB, sul1*, *sul2, bla*_PER-7_*, cmlA5, arr-2*	-	-	*ABCX-DUF262-ZL*	-	+	CP072276
p1KSK10	KSK10	218	622	2020	India	Respiratory specimen	*mph-msr(E), armA, strAB, sul1*, *sul2, bla*_PER-7_*, cmlA5, arr-2*	-	-	*ABCX-DUF262-ZL*	-	+	CP072281
p1KSK11	KSK11	218	622	2020	India	Respiratory specimen	*mph-msr(E), armA, strAB, sul1*, *sul2, bla*_PER-7_*, cmlA5, arr-2*	-	-	*ABCX-DUF262-ZL*	-	+	CP072286
p1KSK18	KSK18	218	622	2020	India	Respiratory specimen	*mph-msr(E), armA, strAB, sul1*, *sul2, bla*_PER-7_*, cmlA5, arr-2*	-	-	*ABCX-DUF262-ZL*	-	+	CP072291
p1KSK19	KSK19	218	622	2020	India	Respiratory specimen	*mph-msr(E), armA, strAB, sul1*, *sul2, bla*_PER-7_*, cmlA5, arr-2*	-	-	*ABCX-DUF262-ZL*	-	+	CP072296
p1KSK20	KSK20	218	622	2020	India	Respiratory specimen	*mph-msr(E), armA, strAB, sul1*, *sul2, bla*_PER-7_,*cmlA5, arr-2*	-	-	*ABCX-DUF262-ZL*	-	+	CP072301
pA1429c	A1429	205	108	2010	China	Secretion	*blaTEM-1B, aacC2, aacA1, strAB, sul2, tetA*(B)	-	+	+	*puuE, uao, hiuH, uacT, (*ISAha2*), allA, alc, hpxO*^d,e^	-	CP046899
pA297-3	A297(RUH875)	200	1	1984	Netherlands	Urinary tract infection	*strAB, sul2*	-	+	*ABCX-DUF262-ZL*	*puuE, uao, hiuH, uacT, (*ISAha2*), allA, alc, hpxO*	+	KU744946
pAB04-1	Ab04-mff	169	10	2012	Canada	Blood	*mph-msr(E), armA, strAB, sul1*, *sul2*, *bla*_PER-7_*, cmlA5, arr-2, tetA*(B)	-	-	-	-	-	CP012007
pAB3	ATCC 17978-mff	148	437	2014	Canada	NA^f^	*sul2*	-	-	-	-	-	CP012005
pAb45063_b	Acb-45 063	183	NA	NA	NA	NA	*strAB, sul2*	*B∆CDE* ^g^	+	+	*puuE, uao, hiuH, uacT*	-	MK323043
pAba7804b	7804	170	25	2006	Mexico	Bronchoalveolar lavage fluid	*strAB, sul2, tetA*(B)	*ABCDEΔ*	-	+	-	+	CP022285
pB11911	B11911	216	149	2014	India	Blood	*mph-msr(E), armA, strAB, sul1*, *sul2, bla*_PER-7_*, cmlA5, arr-2*	-	-	*ABCX-DUF262-ZL*	-	+	CP021344
pD4	D4	132	25	2006	Australia	NA	*strAB, sul2*	-	-	-	-	-	KT779035
pD46-4	D46	207	25	2010	Australia	NA	*mph-msr(E), strAB, sul2, tetA*(B)	-	+	*ABCX-DUF262-ZL*	*puuE, uao, hiuH, uacT*	+	MF399199
pHWBA8_1	HWBA8	195	25	2013	South Korea	Sputum	*mph-msr(E), armA, aacA1, aacC2, sul1*, *sul2*, *bla*_PER-7_*, cmlA5, arr-2, tetA*(B)	*DΔE*	-	+	-	-	CP020596
pIOMTU433	IOMTU433	189	622	2013	Nepal	NA	*mph-msr(E), armA, strAB, sul1*, *sul2, bla*_PER-7_,*cmlA5, arr-2*	-	-	*ABCX-DUF262-ZL*	-	+	AP014650
p2NCTC7364^h^	NCTC7364	148	494	NA	NA	NA	*sul2*	-	-	-	-	-	LT605060
pMC1.1	MC1	184	991	2015	Bolivia	Catheter	*aacC2, aacA1, strAB, sul2, tetA*(B)	-	*DAΔCPTR*	*AΔBΔCΔXΔZΔLΔ*	*puuE, uao∆, hiuH, uacT∆*	∆	MK531536
pMC75.1	MC75	150	15	2016	Bolivia	Ulcer	*strAB, sul2*	-	+	+	*puuE, uao, hiuH, uacT*	-	MK531540
pPM193665_1	PM193665	150	10	2019	India	Pus	*mph-msr(E), armA, strAB, sul1, sul2*, *ble-MBL, blaNDM-1, cmlA5, arr-2, tetA*(B)	-	-	-	-	-	CP050416
pPM194122_1	PM194188	150	10	2019	India	BAL	*mph-msr(E), armA, strAB, sul1, sul2*, *ble-MBL, blaNDM-1, cmlA5, arr-2, tetA*(B)	-	-	-	-	-	CP050426
pPM194229_1	PM194229	226	10	2019	India	BAL	*mph-msr(E), armA, strAB, sul1*, sul2, *bla*_PER-7_*, cmlA5, arr-2, tetA*(B)*)*	-	+	*ABCX-DUF262-ZL*	*puuE, uao, hiuH, uacT, (ISAha2), allA, alc, hpxO∆*	+	CP050433
pVB82_1	VB82	215	25	2019	India	Blood	*mph-msr(E), aacA1, armA, strAB, sul1*, *sul2*, *blaOXA-23, bla*_PER-7_*, cmlA5, arr-2, tetA*(B)	*DΔE*	-	+	-	-	CP050386
p1AR_0088^h^	AR_0088	146	25	NA	NA	NA	*aacC2, aacA1, strAB, sul2, tetA*(B)	*DΔE*	-	+	-	-	CP027531
p1P7774^h^	P7774	202	25	2018	India	Pus	*mph-msr(E), armA, strAB, aacA1, sul1*, *sul2*, *bla*_PER-7_*, cmlA5, arr-2, tetA*(B)	*DΔEΔ*	-	*ABCΔXZL*	-	-	CP040260
p1VB16141^h^	VB16141	189	622	2019	India	Blood	*mph-msr(E), armA, strAB, sul1*, *sul2, bla*_PER-7_*, cmlA5, arr-2*	-	-	*ABCXΔ-DUF262-ZL*	-	+	CP040051
p1VB35179^h^	VB35179	236	1512	2018	India	Blood	*mph-msr(E), armA, strAB, sul1*, *sul2*, *bla*_PER-7_*, cmlA5, arr-2, tetA*(B)	-	+	*ABCX-DUF262-ZL*	*puuE∆, uao, hiuH∆, uacT, (*ISAha2*), allA, alc, hpxO*	+	CP040054
p40288^h^	40 288	145	25	2021	France	Urine	*aacC2, aacA1, strAB, sul2, tetA*(B)	*DΔE*	-	+	-	-	CP077802
pR32_1	nord4-2	117	25	2022	Germany	NA	*strAB, sul2*	-	-	-	-	-	CP091598
pAB5	UPAB1	100	25	2019	Argentina	Urine	*strAB, sul2, tetA*(B)	-	-	-	*puuE, uao, hiuH, uacT*	-	CP032216
pCl107	Cl107	198	25	2012	Lebanon	Urine	*aacC2, aacA1, strAB, sul2, tetA*(B)	+	+	+	*puuE, uao, hiuH, uacT*	+	CP098522

a + or—means the complete presence or absence of the module. When the module is incomplete, the left genes are written.

b *CYP* for cytochrome P450 encoding gene. All P450 are 100% identical to GenPept acc. no. WP_001089493.1 except for pCL107, pAba7804b and pMC1.1 that are 99%-100% identical to GenPept acc. no. WP_004658516.1 and the P450 of VB35179 plasmid is 84% similar to GenPept acc. no. WP_001089493.1. The CYP of pMC1.1 is disrupted.

c DUF262 corresponds to DUF262 domain containing HNH endonuclease family protein encoding gene.

d *puuE* for allantoinase, *uao* for OHCU decarboxylase, *hiuH* for hydroxyisourate, *hpxO* for urate oxidase, *alC* for allantoicase and *allA* for ureidoglycolate hydrolyase encoding genes.

e The inserted insertion sequences within the module is enclosed within parentheses.

f not available.

g Δ indicates incomplete, interrupted, or frameshifted genes.

h named here.

### pCl107 contains a resistance region that serves as a missing link in the evolution of AbGRI1 antibiotic resistance islands

**Figure 1.**
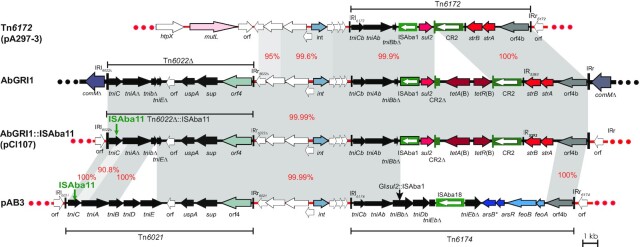
Comparison of AbGRI1 and resistance regions found in pA297-3, pCl107, and pAB3. Red horizontal lines represent plasmid backbones and the chromosome of global clone 2 (GC2) is shown by a thick line with the *comM* gene present on either side of AbGRI1. Arrows represent the extent and orientation of genes or orfs, with their names shown below. Resistance genes are in red. The extents of Tn*6021*, Tn*6022*, Tn*6172*, and Tn*6174* are shown below or above the genomic region. Vertical bars at the ends of the elements indicate their IRs. Different shades of grey join regions with significant DNA identities, with identities shown by red numbers. ISAba1, ISAba18 and CR2 are shown using a green box with a white arrow indicating the direction of the *tnp* gene. ISAba11 and GI*sul2*:: ISAba1 are shown above their location. The GenBank acc. no. for pA297-3, pCl107, and pAB3 are KU744946, CP098522, and CP012005, respectively. The scale bar is shown.

The *strAB* genes, together with *sul2* and *tetAR*(B), were found in pCl107 within a variant of Tn*6172*, which was previously described in pA297-3 (GenBank acc. no. KU744946) with an additional CR∆-*tetAR*(B) segment (compared to Tn*6172*) (Fig. 1). We previously presented a detailed analysis of the resistance regions found in pA297-3 and pAB3 (GenBank acc. no. CP012005). We proposed that the ancestral structure of AbGRI1 island (AbGRI1-0), which is frequently found in the chromosomal *comM* genes of GC2 isolates, has evolved from a close relative of pAB3 with Tn*6022* in place of Tn*6021* and in which Tn*6174* had already evolved to form Tn*6172* (Hamidian and Hall 2017).

Here, analysis of the surrounding regions of the Tn*6172*-var in pCl107 showed the presence of a Tn*6022∆*:: ISAba11 precisely where Tn*6022∆* would be, relative to Tn*6172*-var, in AbGRI1 variants (Fig. 1). In pCl107, the ISAba11 copy interrupts the *tniC* gene of the Tn*6022∆* that is missing the *tniD*, and parts of the *tniE* and *tniB* genes. Notably, while the sequence of this Tn is identical to Tn*6022*, the ISAba11 copy is present precisely where an ISAba11 copy is also present in Tn*6021* in pAB3 (Hamidian and Hall 2017). The Tn*6022∆*:: ISAba11 structure found in pCl107 shares the ISAba11 position with pAB3 and the sequence of Tn*6022* with AbGRI1 islands, indicating that the ISAba11 insertion is an earlier event. Tn*6021* in pAB3 has further diverged from Tn*6022* by the acquisition of a short DNA spanning the 3'-end of *tniC* and 5'-end of *tniA* from an unknown source via homologous recombination. It also indicates that pCl107 diverged differently by a deletion in the *tniDE* region before it moved to GC2s (prior to the acquisition of ISAba11). Putting together all this information, the resistance region found in pCl107 serves as an important missing link in the complex evolutionary history of AbGRI1 islands as it provides further support for the theory that this segment has transposed as a single unit into the *comM* gene of the ancestor of the GC2 clone.

In addition to the *strAB*-*sul2*-*tetA*(B) region, another resistance cluster region encompassing *aacC2* and *aacA1* genes was found in pCl107. Indeed, these genes are absent in pD4, pA297-3 and pD46-4 (GenBank acc. no. KT779035, KU744946, MF399199 respectively) but present in several other *A. baumannii* plasmids such as pMC1.1, p1AR_0088, pA1429c, pHWBA8_1, and p40288 (GenBank acc. no. MK531536, CP027531, CP046899, CP020596, CP077802, respectively) (Table 1). The two aminoglycoside resistance genes are surrounded by several complete and truncated mobile elements, including two CR1∆ copies, an ISKpn11Δ, an IS*1008*, and an IS*1006*∆. In the absence of an ancestral structure in GenBank, it was not possible to determine the evolution of this region. Hence, further sequence data will be required to show how this complex resistance region has evolved.

pCl107 has an intact *merDACPTR* module,>99% DNA identical to that carried by pA297-3. This mercury resistance module is present in many plasmids, as described previously (Hamidian, Ambrose and Hall 2016) (Table 1). However, it is absent the pD4.

### pCl107 encodes a type 1 BacteRiophage Exclusion (BREX) system

**Figure 2.**
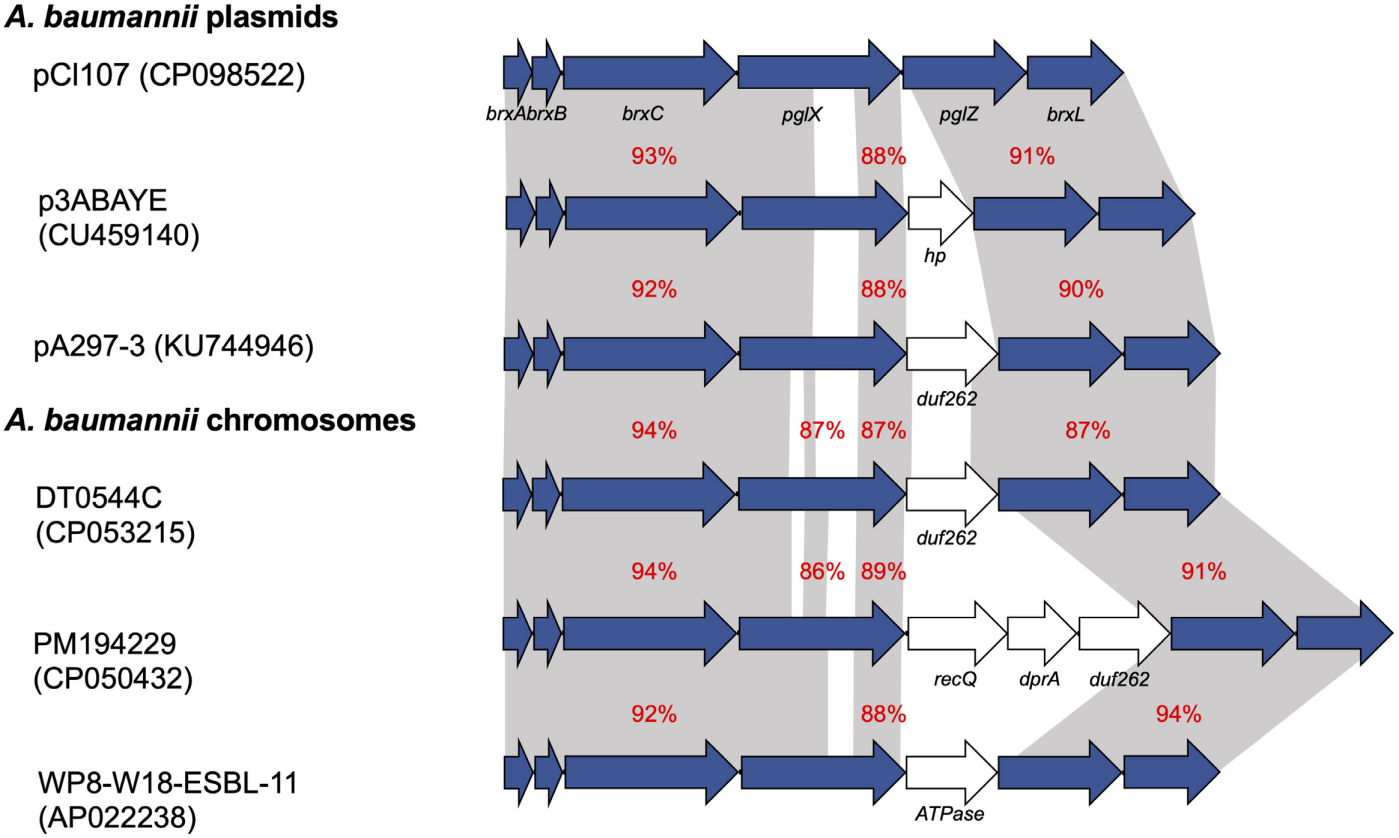
Complete BREX structures, related to the pCl107 BREX module, found in *A. baumannii* plasmids and chromosomes. The BREX genes are shown using blue arrows. Other genes are shown using white arrows. *duf262* encodes a DUF262 domain-containing HNH endonuclease family protein, *dprA* encodes a DNA-processing protein DprA, *recQ* codes for a RecQ family ATP-dependent DNA helicase, and *hp* encodes a hypothetical protein. The figure is drawn to scale.

The pCl107 has a BREX (BacteRiophage Exclusion) system located at bases 125 913 to 139 090 (of GenBank acc. no. CP098522). BREX system has recently been described as a phage resistance system and added to the previously known mechanisms (CRISPR-Cas and restriction-modification systems) in the arsenal of bacterial defence against phages (Goldfarb et al. 2015, Hampton, Watson and Fineran 2020). The BREX system includes at least six subtypes, each with a different gene component and operon organization (Goldfarb et al. 2015). Our analysis showed that the BREX system carried by pCl107 belongs to Type 1 as it includes all six genes (*brxABC, pglXZ, and brxL*) that are characteristic of this type (Fig. 2). This type shares two genes, *pglX* encoding an adenine-specific DNA methyltransferase and *pglZ* encoding an alkaline phosphatase, with the *pgl* system, which was first identified in *Streptomyces coelicolor* (Goldfarb et al. 2015). The other four genes encode a Lon-like protease (*brxL*), an-ATP binding protein (*brxC*), an unknown protein (*brxB*), and NusB-like RNA binding antitermination protein (*brxA*).

A previous study has shown that BREX and BREX-like systems are distributed in about 10% of microbial genomes (Goldfarb et al. 2015). Although BREX has been reported in plasmids of *Acinetobacter bereziniae* (Brovedan et al. 2019) and *A. baumannii* (Cerezales et al. 2020), no comparative analysis has been done. To identify BREX systems related to that found in pCl107, the GenBank non-redundant database was searched using the DNA sequence of this region (from pCl107) as a query. Several matches were found including 36 *A. baumannii* plasmids either belonging to pCl107 (26) or unrelated (10), fourteen *A. baumannii* chromosomes, and several other entries from *Acinetobacter* spp., listed in Table S3, were found. These BREX modules differ by the presence and absence of additional genes between *pglZ* and *pglX* (Fig. 2, Table S3), which can differentiate the three BREX gene clusters found in *A. baumannii* plasmids as well as those in *A. baumannii* chromosomes (Fig. 2).

**Figure 3.**
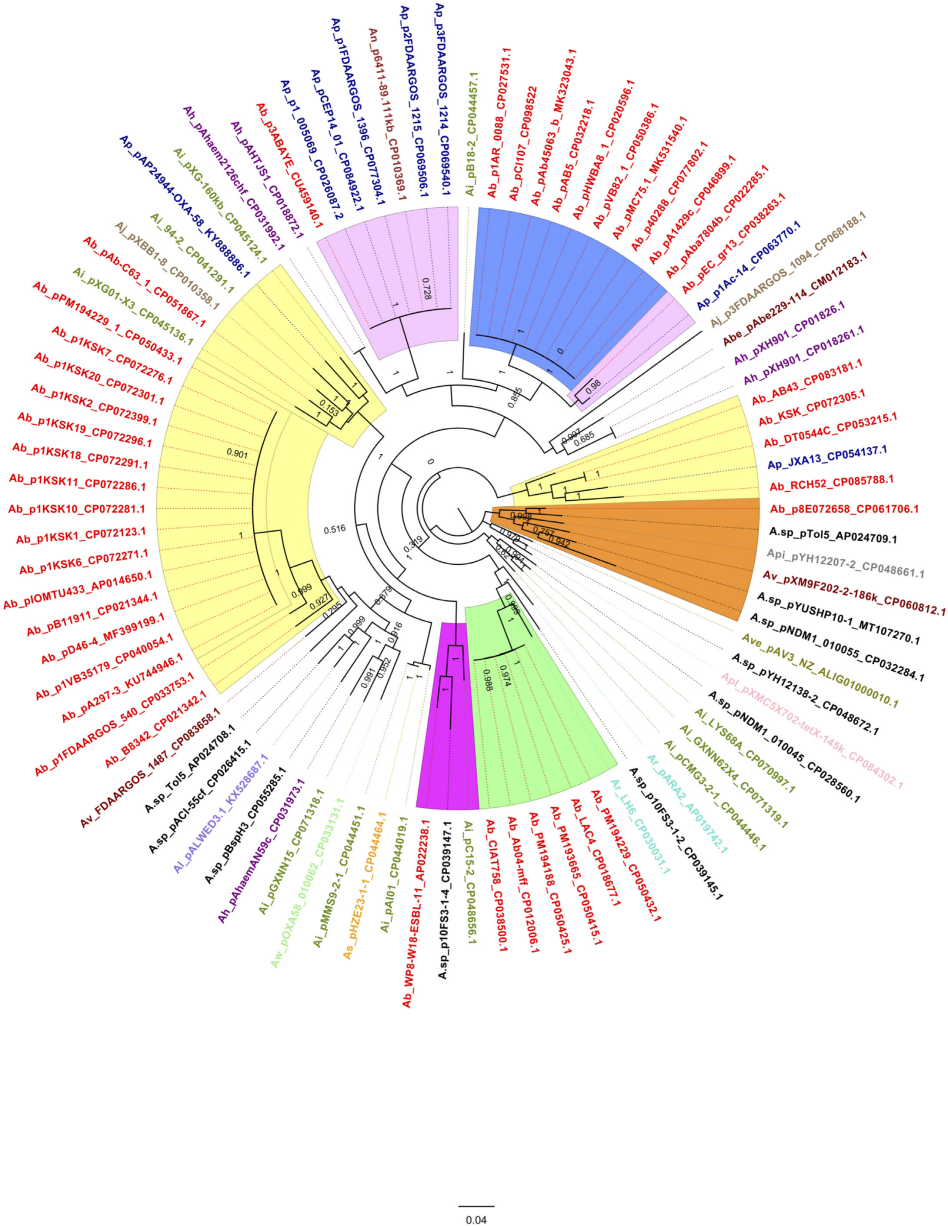
Maximum likelihood phylogenetic tree of BREX sequences drawn using the FastTree software. The phylogenetic tree was constructed using the DNA sequence of 90 BREX regions from *brxA* to *brxL*. The clusters encompassing *A. baumannii* are highlighted according to the nature of the intruding gene. The clusters with no intruding gene are highlighted in blue (enclosing the pCl107) or orange (without pCl107). The yellow and pink clusters contain one intruding gene encoding the DUF262 domain containing the HNH endonuclease family and hypothetical protein, respectively. The green cluster includes the BREX structure with three intruding genes. The first two or three letters of different taxa names include an abbreviation of genus and species of each entry followed by their plasmid/chromosome names (those start with letter p refere to plasmid names and other are chromosome/strain names) and GenBank accession numbers. The abbreviations of species names are: Ab for *A. baumannii* (red), Ap for *A. pittii* (Dark blue), An for *A. nosocomialis* (brown), Aj for *A. johnsonii* (pale brown), As for *A. schindleri* (orange), Ai for *A. indicus* (bitter lime), A. sp for *Acinetobacter* spp. (black), Ar for *A. radioresistens* (turquoise), Ah for *A. haemolyticus* (purple), Al for *A. lwofii* (medium purple), Apl for *A. pseudolwofii* (pink), Api for *A. piscicola* (grey), Aw for *A. wuhouensis* (light green), Av for *A. variabilis* (dark red), Abe for *A. bereziniae* (maroon), Ave for *Acinetobacter venetianus* (olive). Shimodaira-Hasegawa local support values ranged from 0 to 1.

The phylogenetic analysis of BREX regions revealed the presence of many clusters where BREX loci with similar arrangements and identities generally clustered together (Fig. 3). For instance, the BREX gene clusters found in *A. baumannii* include a DUF262 domain-containing HNH endonuclease family protein encoding gene between *pglZ* and *pglX*, which is clustered in three different subgroups (Fig. 3) where each cluster has different DUF262 amino acid identities: identity 65% between those of pA297-3 and pAB_C63_1 (GenBank acc. no. KU744946 and CP051867, respectively) and 59% between those of pA297-3 and DT0544C chromosome (GenBank acc. no. KU744946 and CP053215 respectively).

We found 24 plasmids related to pCl107 that include a complete BREX gene cluster. Two gene clustering patterns were generally identified—one group (15 plasmids including pA297-3) included the DUF262 domain-containing HNH endonuclease family protein-encoding gene, and the second group (with nine plasmids including pCl107) did not have this gene (Table 1, Fig. 3). The DNA identities between BREX pCl107 and pA297-3 ranges between 88% to 96%, indicating thus the presence of two different pathways for the evolution within the plasmids’ BREX and perhaps different impact on immunity against phages (Fig. 3).

Many clusters delimited by the phylogenetic tree are diverse in species and genetic backgrounds (Fig. 3). For instance, the *A. baumannii* plasmid BREX Region (pAb-C63_1) clusters together with BREX regions from *Acinetobacter johnsonii, A. indicus*, and *A. pittii*. This indicates the extent to which horizontal gene transfer is possibly taken between *Acinetobacter* spp. and even between plasmid and chromosomal genomes to mitigate the phage pressure.

### The *ptxABCDE* phosphonate metabolism module

**Figure 4.**
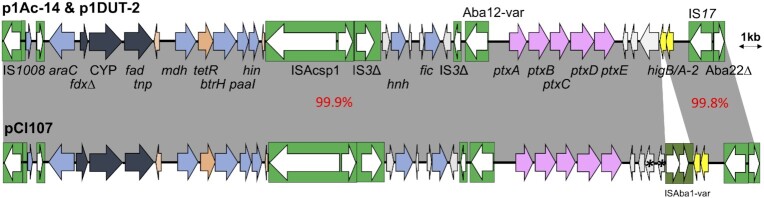
Genetic context of the *ptx* module in pCl107, and *Acinetobacter pittii* plasmids (p1Ac-14 and p1DUT-2). The transposase genes are white arrows enclosed in green boxes. Genes encoding hypothetical proteins are coloured in light grey. The *ptxABCDE* cluster is in rose and the cytochrome P450 cluster is in dark grey. Other genes with known functions are indicated either by blue or shaded orange or yellow arrows according to their nature. The hypothetical gene next to ISAba1-var is disrupted in pCl107 and divided into parts (arrows with star inside) whereas it is intact in other plasmids. The figure is drawn to scale. The GenBank accession numbers for pCl107, p1Ac-14, p1DUT-2 are CP098522, CP063770, and CP014652 respectively.

Phosphorus is an essential component of cell molecules, metabolism, and structure, predominantly present in nature in its most oxidized state as inorganic phosphate (pi) and which is also directly incorporated into biological molecules (Bisson et al. 2017). For instance, in *Escherichia coli* there are more than 30 genes in a regulon called PHO to sense, acquire and process phosphate (Stasi, Neves and Spira 2019). Under phosphate limitations, bacteria can acquire phosphorus by metabolizing reduced phosphorus compounds. Cl107 does not include the *phnCDEFGHIJKLMNOP* operon, which is present in *E. coli* and involved in phosphonate transport and assimilation via C-P lyase complex, however, instead, it contains a putative cluster (*phnXW*-*HpnW-*ABC transporter and regulator encoding genes located at bases 2 485 529–2 493 621) for transporting and processing the 2-aminoethylphosphonate (2-AEPn) which is one of the most widely occurring phosphonates in nature (Borisova et al. 2011, Zangelmi et al. 2021). Interestingly, an additional cluster potentially linked to phosphate metabolism (*ptx* module) was also found in pCl107. This *ptx* module consists of five genes, namely *ptxABCDE*, that are related to those found in *Pseudomonas stutzeri* (amino acid identities for PtxA: 59%, PtxB: 60%, PtxC: 64%, PtxD: 53%, PtxE: 38%)*. ptxABC* encodes an ABC transporter system where PtxA (IPR012693 family with IPR003439 and IPR003593 domains) is an ATP-binding protein, PtxB (IPR005770 family with PF12974 domain) is a periplasmic-binding protein and PtxC (IPR005769 family with domain IPR000515) is a membrane-spanning protein. *ptxD* encodes a phosphonate (traditionally named phosphite) dehydrogenase (IPR006140 and IPR006139 domains) and *ptxE* encodes a transcriptional regulator (IPR005119 and IPR000847 domains) belonging to the LysR family. Additionally, the Cl107 PtxB has the same phosphite-specific cap residues as the PtxB family that are not found in other homologs as C-P lyase-linked phosphonate binding PhnD of *E. coli* and HtxB (part of HtxBCDE hypophosphite transporter), highlighting a potential role for the Cl107 PtxABC as a phosphonate (traditionally named as phosphite) transporter (Fig. S1) (Bisson et al. 2017). GenBank was searched to find *ptx* modules closely related to that found in pCl107. Eighteen (n = 18) plasmids were found to carry either complete or partial segments of the *ptx* module found in pCl107. Seven were in *A. baumannii* plasmids related to pCl107 (Table 1), 5 in *A. pittii*, and 6 in *Acinetobacter* spp. plasmids (Table 2). Notably, all the *ptx* modules found in *A. baumannii* plasmids are incomplete (Table 1). Amongst others, the *ptx* module is found in two *A. pittii* plasmids (p1DUT-2 and p1Ac-14 GenBank acc. no. CP014652 and CP063770, respectively) are identical to that in pCl107 (Fig. 4). Given that most strains containing *ptx* modules were recovered in environmental samples suggests a potential environmental source for this *ptx* module and that it has also been introduced to clinical strains by plasmids related to pCl107 (Table 2).

**Table 2.** Properties of strains with plasmids containing the *ptx* module.

Species name	Strain name	Plasmid name	Size (kb)	Year	Country	Source	ST^IP^	*ptxABCDE*	aa %identity range ^a^	GenBank acc. no.
*A. baumannii*	7804	pAba7804b	170	2006	Mexico	Bronchoalveolar lavage fluid	25	*ABCDE∆* ^b^	100	CP022285
*A. baumannii*	Ab45063_b	pAb45063_b	183	NA^c^	NA	NA	NA	*B∆CDE*	100	MK323043
*A. baumannii*	P7774	p1P7774^d^	202	2018	India	Pus	25	*DΔEΔ*		CP040260
*A. baumannii*	HWBA8	pHWBA8_1	195	2013	South Korea	Sputum	25	*DΔE*	100	CP020596
*A. baumannii*	AR_0088	p1AR_0088^d^	146	NA	NA	NA	25	*DΔE*	100	CP027531
*A. baumannii*	VB82	pVB82_1	215	2019	India	Blood	25	*DΔE*	100	CP050386
*A. baumannii*	40 288	p40288c	145	2015	France	Canis^e^ lupus/urine	25	*DΔE*	100	CP077802
*A. pittii*	DUT-2	p1DUT-2^d^	141	2015	China	Marine sediments	640	*ABCDE*	100	CP014652
*A. pittii*	Ac-14	p1Ac-14^d^	117	2016	China	Fruit tree rhizosphere soil	640	*ABCDE*	100	CP063770
*A. pittii*	2014S07-126	p2014S07-126-3	32	2014	Taiwan	Homosapiens/NA	64	*A∆B∆C∆D∆E∆*		CP033533
*A. pittii*	WP2-W18-ESBL-11	pWP2-W18-ESBL-11_1	87	2018	Japan	Wastewater treatment plant effluent	1606	*ABCDE*	97–100	AP021937
*A. pittii*	BEC1-S18-ESBL-01	pBEC1-S18-ESBL-01_1	88	2018	Japan	Oceanic water	457	*ABCDE*	97–100	AP022303
*A. haemolyticus*	2126ch	pAhaem2126chf	70	2011	Mexico	Ascites liquid	NA	*ABCDE*	98–100	CP031992
*A. haemolyticus*	TJS01	pAHTJS1	56	2012	China	Homosapiens/NA	NA	*ABΔCΔDE*	98–99	CP018872
*A. haemolyticus*	AN4	pAhaemAN4d	107	2010	Mexico	Ulcer by decubitus	NA	*ABCDE*	97–100	CP031980
*A. radioresistens*	FDAARGOS_731_3	pFDAARGOS_731_3	61	NA	USA	Normal skin of the right arm	NA	*ABCDE*	99–100	CP059688
*A. bereziniae*	GD0320	pGD0320	122	2016	China	Sputum	NA	*ABCDE*	98–100	CP066122
*A. defluvii*	WCHA30	pOXA58_010 030	355	2015	China	Sewage	NA	*ABCDE*	99–100	CP029396

a compared to Ptx proteins encoded by pCl107. Only complete proteins are compared.

b Δ indicates incomplete, interrupted, or frameshifted genes.

c not available.

d named here by preceding the strain name by the letter *p*.

e host is only indicated in the source column if the clinical sample is not obtained from the human host or its exact origin is unknown.

### pCl107 encodes a class B cytochrome P450

Additionally, in pCl107, a class B cytochrome P450 with three components: a FAD-containing flavoprotein ‘NAD(P)H-dependent reductase’ (domains: IPR028202, IPR023753), P450 (families: IPR002397, IPR001128), and an iron-sulphurs protein (family: IPR001055; domain: IPR001041) is present in the vicinity of the *ptx* module (Fig. 4). Although found in organisms across the tree of life, cytochrome P450 enzymes (CYP or P450s) have attracted particular interest primarily for their potential usages in bioremediation, where bacterial P450 often oxidize environmental chemicals to ease their catabolism (Behrendorff 2021). These enzymes are classified into several families and subfamilies (Nelson 2006). The class B cytochrome P450 encoded by pCl107 is a 463 aa identical to that of *Moraxellaceae* P450 (e.g. GenPept accession number WP_004 658 516), which belongs to the family 153 of the subfamily A, which is also 71% aa identical to CYP153A1 of *Acinetobacter calcoaceticus* (https://drnelson.uthsc.edu/bacteria/). In plasmids related to pCl107, all the P450 identified belong to the same family, with the majority 99% identical to the *A. calcoaceticus* P450 and only two almost identical to pCl107 P450 (Table 1). Indeed, CYP153 proteins are known to encode alkane hydroxylases able to degrade short- and medium-chain-length n-alkanes and are often retrieved in petroleum-contaminated soil, groundwater, and coastal seawater, adding more shreds of evidence on the potential environmental ancestor of pCl107 (Wang et al. 2010, Nie et al. 2014). Similarly, a recent study highlighted that the bacterial lifestyle in Gammaproteobacteria species shaped to such an extent the P450 repertoire, where the pathogenic or commensal lifestyle is associated with the lowest number of P450s (Msomi et al. 2021).

### Uric acid module

**Figure 5.**
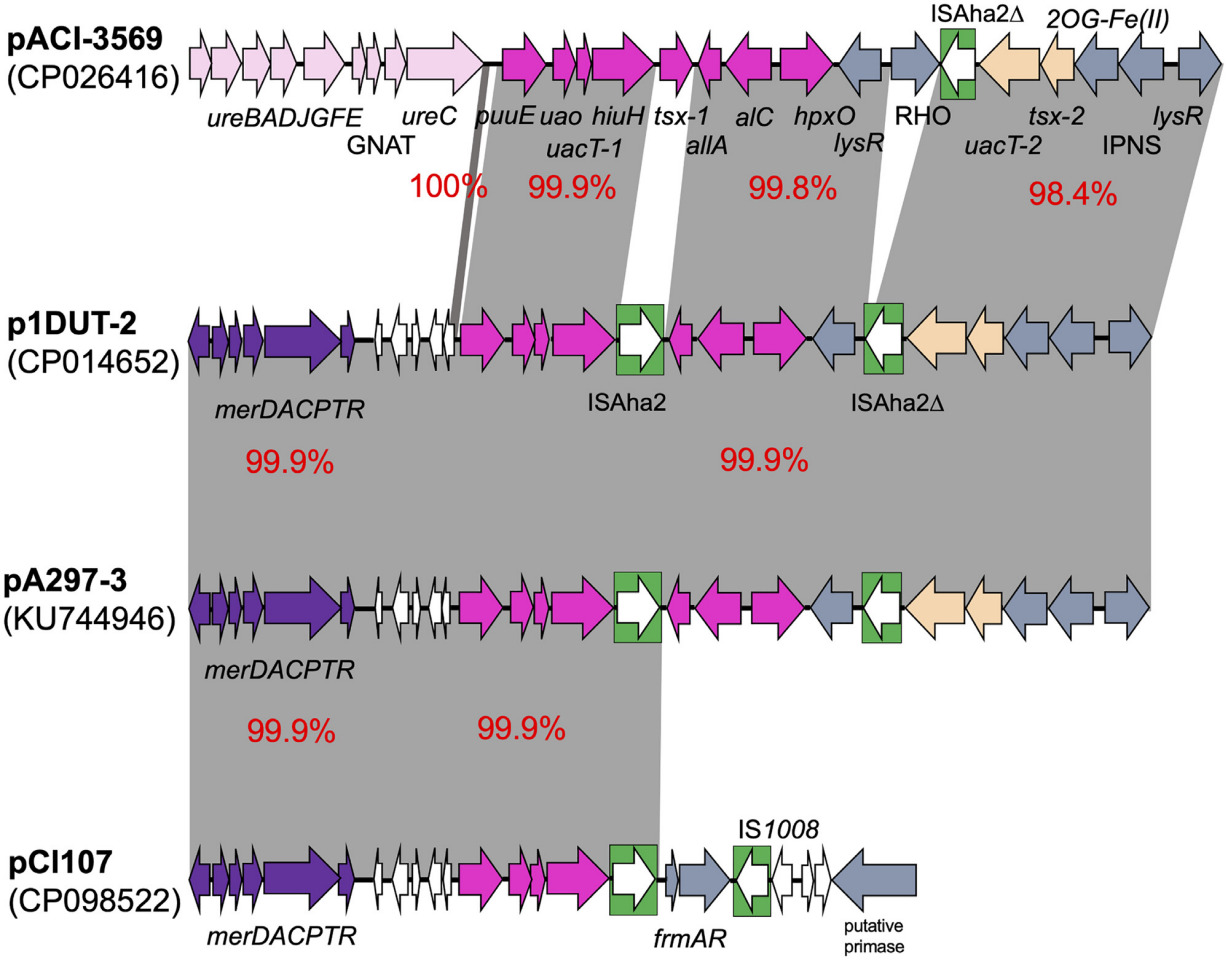
Uric acid catabolism module in pACI-356, p1DUT-2, pA297-3, pCl107. The transposase genes are white arrows enclosed in green boxes. White genes encode hypothetical proteins. The uric acid module is coloured in pink, whereas the urease module in light pink and mercuric module in purple. Other genes with known functions are indicated by grey arrows. The genes likely involved in purine metabolism are coloured in light orange. The genes *uacT* for uric acid transporter encoding gene, *hiuH* for hydroxyisourate hydrolase encoding gene, *uao* for 2-oxo-4-hydroxy-4-carboxy-5-ureidoimidazoline decarboxylase encoding gene, *puuE* for allantoinase encoding gene, *allA* for ureidoglycolate lyase encoding gene, *alC* for allantoicase encoding gene.

Four genes (*puuE, uao, hiuH, uacT*) encoding uric acid metabolism and transport functions are also carried by pCl107 (Fig. 5). The *uacT* gene encodes a permease that belongs to the xanthin.uracile permease family proteins (IPR006042 and the UacT-like: IPR017588). The *hiuH* and *uao* genes encode an hydroxyisourate (HIU) hydrolase (family: IPR014306) and 2-oxo-4-hydroxy-4-carboxy-5-ureidoimidazoline (OHCU) decarboxylase (domain: IPR017595) respectively. The HiuH and Uao proteins are responsible for the second (hydrolysis of HIU to OHCU) and third (the decarboxylation of OHCU to (S)-allantoin) steps of uric acid processing, respectively (Lee et al. 2013). The *puuE* gene is an allantoinase encoding gene catalyzing the hydrolysis of (S)-allantoin to allantoate. While these three encoded enzymes can potentially take an HIU and transform it to allantoate, the urate oxidase (also known as HpxO in *Acinetobacter* (Michiel et al. 2012)) encoding gene responsible for the first step of uric acid catabolism is missing, likely due to an ISAha2-mediated deletion event in pCl107. Hence, the pCl107 uric acid module is likely unable to hydroxylate urate into HIU.

To find plasmids that can potentially catabolize uric acid, we searched 616 complete *A. baumannii* plasmids that were publicly available in the GenBank non-redundant database as of September 2021. Nine plasmids (related to pCl107 carried by *A. baumannii* strains) were found, including four plasmids with the *hpxO* gene and five plasmids without *hpxO* (Table 1 and S4). Among those plasmids with the *hpxO* gene, pA297-3 and pA1429c (GenBank acc. no. KU744946 and CP046899, respectively) include a related sequence (DNA identity 99%) with a complete set of uric acid module genes except for the *tsx*, which is replaced by ISAha2 (Fig. 5).

Additionally, several partial uric acid modules related to that in pCl107 (*puuE-uao-hiuH-uacT*) were found in multiple *Acinetobacter* spp. chromosomes and plasmids (Table S5). Among *Acinetobacter* spp. plasmids, p1DUT-2 (GenBank acc. no. CP014652) shares a similar module and surroundings to pA297-3 with 99.9% DNA identities (Fig. 5). Amongst the entries found here, only pACI-3569 and p1FDAARGOS_1092 (GenBank acc. no. CP026416 and CP068202) have uric acid modules almost identical to that in pCl107 (99.9% DNA identity) (Fig. 5). In addition to the *hpxO* gene, those with complete modules also contain genes encoding allantoicase, which catalyzes the degradation of allantoate to urea and ureidoglycolate, and ureidoglycolate hydrolase that degrades ureidoglycolate to glyoxylate and another molecule of urea (Fig. 5). As well, it harbors a *tsx* gene located next to *uacT* gene and encoding a nucleoside-specific channel-forming protein Tsx. These uric acid clusters in those plasmids could represent a more ancestral clusters of p1DUT-2 and pA297-3, which have subsequently given rise to the pCl107 cluster (Fig. 5). It is also notable that the pCl107 uric acid module was found to be closely related to several *Acinetobacter* strains (Table S5); *Acinetobacter lowfii* H7 and Al-065 and *Acinetobacter* spp. ACNIH1 (GenBank acc. no. CP072549, CP078045 and, CP026420 respectively) have DNA identities ranging from 99% to 100%, suggesting a chromosomal origin.

**Figure 6.**
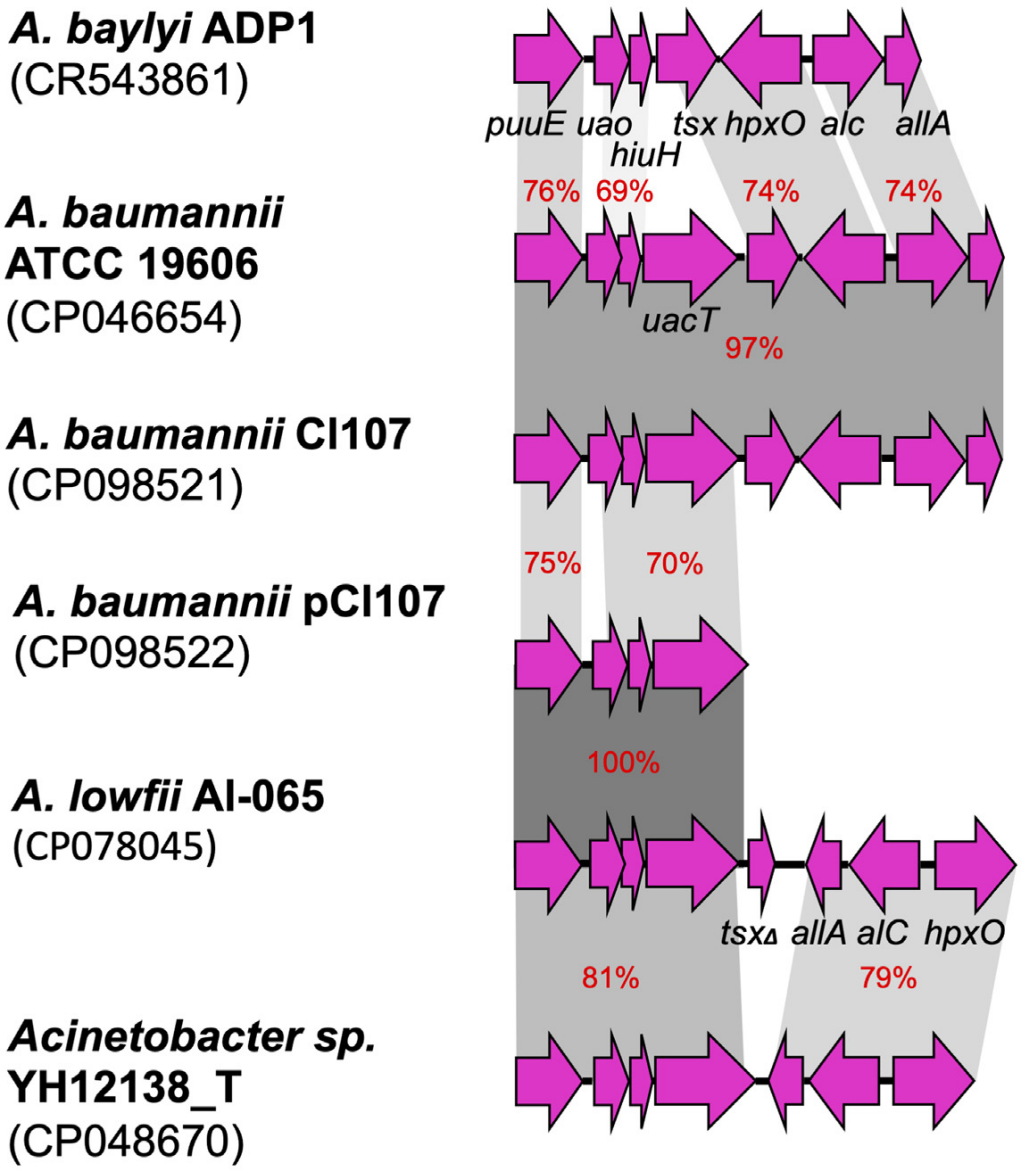
Comparison of uric acid modules found in pCl107 and *A. baumannii* and *Acinetobacter* spp. chromosomal modules. Genes associated with the uric acid module are coloured in pink. Horizontal arrows indicate the extent and orientation of genes.

Cl107 also carries an additional chromosomal gene cluster encoding uric acid catabolism functions. This cluster is distantly related to that in pCl107 (amino acid identity range 56%–80%, Fig. 6), and to that previously characterized (amino acid identity range 58%–84%) in *Acinetobacter. baylyi* (GenPept acc. no. CAG70179-CAG70185) (Michiel et al. 2012) (Fig. 6). In addition, *A. baumannii* module has a unique gene not present in *A. baylyi* module encoding a purine or uric acid permease (UacT) that is homologous (amino acid identities 39%–44%) to PucJK proteins (GenPept acc. no. WP_003243942, WP_003244570), which are involved in uric acid transport in *Bacillus subtilis*.

Uric acid is a potent antioxidant in humans protecting cells from reactive oxygen species. It is released by necrotic mammalian cells as a signal to activate the immune system against microbes. It is well known that bacteria can exploit uric acid as a source of nitrogen and even carbon and energy. However, whether there is any relationship between the presence of uric acid and microbial virulence is poorly understood. In *A. baumannii*, genes associated with purine and pyrimidine biosynthesis have been identified within novel gene clusters required for surface-associated mobility (Blaschke, Skiebe and Wilharm 2021), raising potential roles of purine metabolism-associated and uric acid catabolism genes.

## Conclusion

This study adds another complete genome sequence of an ST25 *A. baumannii* strain recovered in the MENA region in Lebanon and characterizes a large plasmid carried by this strain named pCl107. pCl107 carries a set of antimicrobial resistance genes (*aacA1, aacC2, sul2*, *strAB*, *tetA*(B)) distributed in two complex regions and many non-antimicrobial resistance clusters. We showed that the pCl107 region encompassing the *sul2*, *strAB*, *tetA*(B) is closely related to AbGRI1 islands, commonly found in the chromosomal *comM* gene of GC2 strains, with an addition of the insertion of ISAba11 as the only difference (Fig. 1). This ancestral form of AbGRI1 provides yet another piece of evidence for the transposition of this unit (before it acquired ISAba11) from a plasmid related to pCl107 into the chromosome of an ancestral GC2 strain. This is an important finding highlighting the role of plasmids related to pCl107 in the evolution and acquisition of large genomic islands that carry multiple antibiotic-resistance genes by the most widespread strains of *A. baumannii*, GC2.

pCl107 contains a Type 1 BREX system and constitutes one of the two main evolution patterns observed in BREX clusters found in its related plasmids. This could indicate an association with different impacts on bacterial immunity against phages. pCl107 also carries a *ptx* module for phosphonate metabolism and an incomplete uric acid catabolism module. Here, we untangled the relationships of these regions with several regions in many *Acinetobacter* spp. plasmids and chromosomal and identified possible ancestors for some of the clusters. The *ptx* module found in pCl107 is complete and is likely to be the ancestral structure compared to those found in other related plasmids in ST25 strains. The presence of those environmentally advantageous genetic clusters along with class B cytochrome P450 module in pCl107 and their relatedness with those from environmental isolates could pinpoint the potential environmental ancestor for pCl107.

Plasmids related to pCl107, pD4, and pA297-3 belong to an important class of *Acinetobacter* plasmids that play an important role in multidrug-resistant dissemination as well as regulation of virulence by encoding several regulatory functions interacting with other plasmids and the chromosome (Di Venanzio et al. 2019a, Weber et al. 2015, Hamidian and Hall 2016, Hamidian, Ambrose and Hall 2016, Nigro and Hall 2017, 2019b, Benomar, Di Venanzio, and Feldman 2021, Sycz et al. 2021). These plasmids carry a range of genetic material and complex evolutionary history with many links to multiple antibiotic resistance and metabolic pathways.

## Accession numbers

Complete genome sequence of Cl107 was deposited in GenBank under accession numbers CP098521 (chromosome) and CP098522 (pCl107). Illumina short reads and MinION long reads are also deposited under the Sequence Read Archive (SRA) accession numbers SRR20613520 and SRR20613519, respectively.

## Supplementary Material

xtac027_Supplemental_Files
