# Supplementary material for: Analysis of pCl107 a large plasmid carried by an ST25 Acinetobacter baumannii strain reveals a complex evolutionary history and links to multiple antibiotic resistance and metabolic pathways
Source: FEMS Microbes. 2022 Nov 18;3:xtac027. doi: 10.1093/femsmc/xtac027 (PMC10117892; doi:10.1093/femsmc/xtac027)
Supplement: xtac027_Supplemental_Files [file xtac027_supplemental_files.zip › Table_S1_Supplementary_Data.docx]

**Table S1.** Properties of complete ST25^IP^ Genomes.

| **Strain/plasmid** | **Size (bp)** | **Isolation Source** | **Collection Date** | **Isolation Country** | **CST type ^a^** | **GenBank acc. no.** | **Plasmid Replication proteins ^b^** |
| --- | --- | --- | --- | --- | --- | --- | --- |
| **40288** |  | Urine | 2015 | France | novel |  |  |
| chromosome | 4084922 |  |  |  |  | CP077801 |  |
| p40288^c^ | 145711 |  |  |  |  | CP077802 | - |
| **HWBA8** |  | Sputum | 2013 | South Korea | CST23 (51) |  |  |
| Chromosome | 4077713 |  |  |  |  | CP020597 |  |
| pHWBA8_1 | 195838 |  |  |  |  | CP020596 | - |
| **AR_0088** |  | na^d^ | na | na | CST23 (51) |  |  |
| Chromosome | 4040515 |  |  |  |  | CP027530 |  |
| p1AR_0088^c^ | 146698 |  |  |  |  | CP027531 | - |
| p2AR_0088^c^ | 41087 |  |  |  |  | CP027532 | - |
| **7804** |  | Bronchoalveolar lavage fluid | 2006 | Mexico | novel |  |  |
| Chromosome | 4159217 |  |  |  |  | CP022283 |  |
| pAba7804a | 12381 |  |  |  |  | CP022284 | 59% identical to Aci1 |
| pAba7804b | 170420 |  |  |  |  | CP022285 | - |
| **P7774** |  | Pus | 2018 | India | novel |  |  |
| chromosome | 4143962 |  |  |  |  | CP040259 |  |
| p1P7774^c^ | 202283 |  |  |  |  | CP040260 | Aci9 |
| p2P7774^c^ | 14880 |  |  |  |  | CP040261 | Aci1 |
| p3P7774^c^ | 5464 |  |  |  |  | CP040262 | 57% identical to Aci2 |
| **UPAB1** |  | Urine | 2016 | Argentina | CST24 (48) |  |  |
| chromosome | 3880245 |  |  |  |  | CP032215 |  |
| pAB5 | 100163 |  |  |  |  | CP032216 | - |
| p1UPAB1^c^ | 80061 |  |  |  |  | CP032217 | 44% identical to Aci5 |
| pAB5 | 28582 |  |  |  |  | CP032218 | - |
| pAB5 | 30320 |  |  |  |  | CP032219 | - |
| p3UPAB1^c^ | 16743 |  |  |  |  | CP032220 | - |
| **D4** |  | Wound | 2006 | Australia | CST25 (32) |  |  |
| chromosome | 4173759 |  |  |  |  | CP048849 |  |
| pD4-1 | 2277 |  |  |  |  | CP048850 | 60% identical to Aci5 |
| pD4 | 132632 |  |  |  |  | CP048851 | - |
| **Nord4-2** |  | na | 2018 | Germany | CST28 (55) |  |  |
| chromosome | 4161539 |  |  |  |  | CP091596 |  |
| pR32_1 | 117234 |  |  |  |  | CP091598 | - |
| pR32_2 | 73145 |  |  |  |  | CP091595 | Aci6 |
| pR32_3 | 11378 |  |  |  |  | CP091597 | - |
| **D46** |  | Urine | 2010 | Australia | novel |  |  |
| chromosome | 4027041 |  |  |  |  | CP048131 |  |
| pD46-1 | 6078 |  |  |  |  | CP048132 | - |
| pD46-2 | 8731 |  |  |  |  | CP048133 | Aci1 |
| pD46-3 | 74916 |  |  |  |  | CP048134 | Aci6 |
| pD46-4 | 208004 |  |  |  |  | CP048135 | - |
| **Cl107** |  | Urine | 2012 | Lebanon | CST24 (48) |  |  |
| chromosome | 4056235 |  |  |  |  | CP098521 |  |
| pCl107 | 198716 |  |  |  |  | CP098522 | - |

^a^ CST stands for CRISPR Sequence Type, only the spacers present next to a *cas* cluster are mentioned. Numbers in brackets indicate the number of spacers in each locus.

^b^ Identities indicate protein identities to the closest known match.

^c^ named here.

^d^ not available.
