# Supplementary material for: Analysis of pCl107 a large plasmid carried by an ST25 Acinetobacter baumannii strain reveals a complex evolutionary history and links to multiple antibiotic resistance and metabolic pathways
Source: FEMS Microbes. 2022 Nov 18;3:xtac027. doi: 10.1093/femsmc/xtac027 (PMC10117892; doi:10.1093/femsmc/xtac027)
Supplement: xtac027_Supplemental_Files [file xtac027_supplemental_files.zip › Table_S2_Supplementary_Data.docx]

**Table S2.** antimicrobial susceptibility profile of Cl107

| Antibiotic (symbol) | Disk content | Annular radius (mm) | Annular diameter (mm) | Susceptibility profile^a^ |
| --- | --- | --- | --- | --- |
| Ampicillin/Sulbactam (SAM) | 10/10 µg | 11 | 28 | S^b^ |
| Ampicillin | 25 µg | 7 | 20 | R^c^ |
| Cefotaxime (CTX) | 30 µg | 3 | 12 | R^b^ |
| Ceftazidime (CAZ) | 30 µg | 5 | 16 | I^b^ |
| Ceftriaxone (CR0) | 30 µg | 5 | 16 | I^b^ |
| Imipenem (IPM) | 10 µg | 15 | 36 | S^b^ |
| Meropenem (MEM) | 10 µg | 7 | 20 | S^b^ |
| Amikacin (AK) | 30 µg | 2 | 10 | R^b^ |
| Gentamicin (CN) | 10 µg | 1 | 8 | R^b^ |
| Tobramycin (TOB) | 10 µg | 2 | 10 | R^b^ |
| Spectinomycin (SH) | 25 µg | 3 | 12 | R^c^ |
| Netilmicin (NET) | 30 µg | 3 | 12 | R^c^ |
| Neomycin (N) | 30 µg | 5 | 16 | R^c^ |
| Kanamycin (K) | 30 µg | 2 | 10 | R^c^ |
| Streptomycin (S) | 25 µg | 2 | 10 | R^c^ |
| Rifampicin (RD) | 30 µg | 7 | 20 | R^c^ |
| Trimethoprim (W) | 5 µg | 0 | 0 | R^c^ |
| Sulfamethoxazole (RL) | 100 µg | 0 | 0 | R^c^ |
| Nalidixic acid (NA) | 30 µg | 0 | 0 | R^c^ |
| Ciprofloxacin (CIP) | 5 µg | 0 | 0 | R^b^ |
| Florfenicol (FFC) | 30 µg | 0 | 0 | R^c^ |
| Chloramphenicol (C) | 30 µg | 0 | 0 | R^c^ |
| Tetracycline (TE) | 30 µg | 3 | 12 | I^b^ |

^a^S for Susceptible, I for Intermediate, and R for Resistant

^b^Interpreted according to the 2022 CLSI breakpoints (<https://clsi.org/>)

^c^Interpreted according to the CDS disk diffusion assay (<http://cdstest.net>)
