# Supplementary material for: Analysis of pCl107 a large plasmid carried by an ST25 Acinetobacter baumannii strain reveals a complex evolutionary history and links to multiple antibiotic resistance and metabolic pathways
Source: FEMS Microbes. 2022 Nov 18;3:xtac027. doi: 10.1093/femsmc/xtac027 (PMC10117892; doi:10.1093/femsmc/xtac027)
Supplement: xtac027_Supplemental_Files [file xtac027_supplemental_files.zip › Table_S3_Supplementary_Data.docx]

**Table S3.** Properties of strains with the BREX cluster.

| **Species** | **Strain** | **Year** | **Country** | **Source^a^** | **ST^IP b^** | **Genomic context^c^** | **Size_kb** | **Genomic position of BREX** | **BREX module (*brxABC, pglXZ, brxL*)^d^** | **aa identity range^e^** | **GenBank acc. no.** |
| --- | --- | --- | --- | --- | --- | --- | --- | --- | --- | --- | --- |
| *A. baumannii* | Cl107 | 2012 | Lebanon | Urine | 25 | pCl107 | 198 | 125913..139090 | *ABCXZL* | - | CP098522 |
| *A. baumannii* | 7804 | 2006 | Mexico | Bronchoalveolar lavage fluid | 25 | pAba7804b | 170 | 120589..133765 | *ABCXZL* | 100% | CP022285 |
| *A. baumannii* | Ab45063_b | NA^f^ | NA | NA | NA | pAb45063_b | 183 | 150903..164080 | *ABCXZL* | 100% | MK323043 |
| *A. baumannii* | UPAB1 | 2016 | Argentina | Urine | 25 | pAB5 | 28 | 7521..20698 | *ABCXZL* | 100% | CP032218 |
| *A. baumannii* | P7774 | 2018 | India | Pus | 25 | p1P7774^g^ | 202 | 157949..171127 | *ABCΔXZL*^h^ | 100% | CP040260 |
| *A. baumannii* | HWBA8 | 2013 | South Korea | Sputum | 25 | pHWBA8_1 | 195 | 128019..141196 | *ABCXZL* | 100% | CP020596 |
| *A. baumannii* | AR_0088 | NA | NA | NA | 25 | p1AR_0088^g^ | 146 | 60339..73516 | *ABCXZL* | 99-100% | CP027531 |
| *A. baumannii* | VB82 | 2019 | India | Blood | 25 | pVB82_1 | 215 | 82041..95218 | *ABCXZL* | 100% | CP050386 |
| *A. baumannii* | MC75 | 2016 | Bolivia | Ulcer | 15 | pMC75.1 | 150 | 20576..33753 | *ABCXZL* | 100% | MK531540 |
| *A. baumannii* | 40288 | 2015 | France | Canis lupus/urine | 25 | p40288^g^ | 145 | 36923.. 50100 | *ABCXZL* | 100% | CP077802 |
| *A. baumannii* | A1429 | 2010 | China | Secretion | 108 | pA1429c | 205 | 103263..116440 | *ABCXZL* | 100% | CP046899 |
| *A. baumannii* | MC1 | 2015 | Bolivia | Catheter | 991 | pMC1.1 | 184 | 50041.. 63231 | *A∆B∆C∆X∆Z∆L∆* |  | MK531536 |
| *A. baumannii* | EC | 2018 | Czech Republic | NA | 345 | pEC_gr13 | 128 | 41433..55934 | *ABCX-HP-ZL* | 95-99% | CP038263 |
| *A. baumannii* | E-072658 | NA | Finland | Paper pulp mill | 649 | p8E072658 | 119 | 71983..85176 | *ABCXZL* | 79-99% | CP061706 |
| *A. baumannii* | A297 RUH875 | 1984 | Netherlands | NA | 1 | pA297-3 | 200 | 133702..148849 | *ABCX-DUF262-ZL* | 78-99% | KU744946 |
| *A. baumannii* | VB35179 | 2018 | India | Blood | 1512 | p1VB35179^g^ | 236 | 147376.. 162523 | *ABCX-DUF262-ZL* | 78-99% | CP040054 |
| *A. baumannii* | PM194229 | 2019 | India | BAL | 10 | chr | 3886 | 2707784..2726585 | *ABCX-RecQ, DprA, DUF262-ZL* | 83-99% | CP050432 |
|  |  |  |  |  |  | p1PM194229 | 226 | 82032.. 97179 | *ABCX-DUF262-ZL* | 78-99% | CP050433 |
| *A. baumannii* | D46 | 2010 | Australia | Mid stream urine | 25 | pD46-4 | 207 | 140189..155336 | *ABCX-DUF262-ZL* | 78-99% | MF399199 |
| *A. baumannii* | B11911 | 2014 | India | Blood | 149 | pB11911 | 216 | 103386..118533 | *ABCX-DUF262-ZL* | 78-99% | CP021344 |
| *A. baumannii* | IOMTU 433 | NA | NA | NA | 622 | pIOMTU433 | 189 | 56630..71777 | *ABCX-DUF262-ZL* | 78-99% | AP014650 |
| *A. baumannii* | KSK6 | 2020 | India | Respiratory specimen | 622 | p1KSK6 | 218 | 84111..99258 | *ABCX-DUF262-ZL* | 78-99% | CP072271 |
| *A. baumannii* | KSK1 | 2020 | India | Respiratory specimen | 622 | p1KSK1 | 218 | 84111..99258 | *ABCX-DUF262-ZL* | 78-99% | CP072123 |
| *A. baumannii* | KSK7 | 2020 | India | Respiratory specimen | 622 | p1KSK7 | 218 | 84111..99258 | *ABCX-DUF262-ZL* | 78-99% | CP072276 |
| *A. baumannii* | KSK20 | 2020 | India | Respiratory specimen | 622 | p1KSK20 | 218 | 84111..99258 | *ABCX-DUF262-ZL* | 78-99% | CP072301 |
| *A. baumannii* | KSK2 | 2020 | India | Respiratory specimen | 622 | p1KSK2 | 218 | 84111..99258 | *ABCX-DUF262-ZL* | 78-99% | CP072399 |
| *A. baumannii* | KSK19 | 2020 | India | Respiratory specimen | 622 | p1KSK19 | 218 | 84111..99258 | *ABCX-DUF262-ZL* | 78-99% | CP072296 |
| *A. baumannii* | KSK18 | 2020 | India | Respiratory specimen | 622 | p1KSK18 | 218 | 84111..99258 | *ABCX-DUF262-ZL* | 78-99% | CP072291 |
| *A. baumannii* | KSK11 | 2020 | India | Respiratory specimen | 622 | p1KSK11 | 218 | 84111..99258 | *ABCX-DUF262-ZL* | 78-99% | CP072286 |
| *A. baumannii* | KSK10 | 2020 | India | Respiratory specimen | 622 | p1KSK10 | 218 | 84111..99258 | *ABCX-DUF262-ZL* | 78-99% | CP072281 |
| *A. baumannii* | VB16141 | 2019 | India | Blood | 622 | p1VB1614^g^ | 189 | 87343.. 102489 | *ABCX∆-DUF262-ZL* | 97-99% | CP040051 |
| *A. baumannii* | FDAARGOS_540 | NA | NA | Clinical isolate | 1542 | p1FDAARGOS_54^g^ | 86 | 14802.. 29925 | *ABCX-DUF262-ZL* | 76-99% | CP033753 |
| *A. baumannii* | AYE | NA | NA | NA | 1 | p3ABAYE | 94 | 53839..68427 | *ABCX-HP-ZL* | 77-99% | CU459140 |
| *A. baumannii* | OCU_Ac18 | NA | NA | NA | NA | pOCUAc18-1 | 90 | 45830..63601 | *ABC∆-*ISAba19*-C∆X-DUF262-ZL∆-*ISAba19*-L∆* | 78-99% | AP024803 |
| *A. baumannii* | Ab-C63 | 2016 | Ghana | Sputum | 107 | pAb-C63_1 | 81 | 10771.. 25903 | *ABCX-DUF262-ZL* | 81-99% | CP051867 |
| *A. baumannii* | 64-3985 | 2007 | China | Homo sapiens/NA | NA | pHS35 | 34 | 23303..25204 | *LΔ* |  | KM884819 |
| *A. baumannii* | BJAB0715 | NA | NA | NA | NA | pBJAB0715 | 52 | 15791..17692 | *LΔ* |  | CP003848 |
| *A. baumannii* | 29FS20 | 2017 | China | Feces of duck | 16 | p29FS20-1 | 66 | 47950..49047 | *LΔ* |  | CP044520 |
| *A. baumannii* | PM193665 | 2019 | India | Pus | 10 | chr | 3948 | 283154.. 301955 | *ABCX-RecQ, DprA, DUF262-ZL* | 82-99% | CP050415 |
| *A. baumannii* | CIAT758 | 2018 | India | Blood | 10 | chr | 4017 | 2297517..2316318 | *ABCX-RecQ, DprA, DUF262-ZL* | 82-99% | CP038500 |
| *A. baumannii* | PM194188 | 2019 | India | BAL | 10 | chr | 4002 | 2819489..2838290 | *ABCX-RecQ, DprA, DUF262-ZL* | 82-99% | CP050425 |
| *A. baumannii* | Ab04-mff | 2012 | Canada | Blood | 10 | chr | 3935 | 2793174..2811975 | *ABCX-RecQ, DprA, DUF262-ZL* | 82-99% | CP012006 |
| *A. baumannii* | LAC4 | 1997 | USA | NA | 10 | chr | 3974 | 2520424..2539225 | *ABCX-RecQ, DprA, DUF262-ZL* | 83-99% | CP018677 |
| *A. baumannii* | B8342 | 2014 | India | Blood | 1545 | chr | 3947 | 2714502.. 2729613 | *ABCX-DUF262-ZL* | 78-100% | CP021342 |
| *A. baumannii* | AB43 | 2017 | China | Sputum | 132 | chr | 3854 | 2657592..2676415 | *ABCX-RecQ, DprA, DUF262-ZL* | 79-100% | CP083181 |
| *A. baumannii* | WP8-W18-ESBL-11 | 2018 | Japan | Wastewater treatment plant effluent | 1548 | chr | 3727 | 2768384..2783476 | *ABCX-AAA family ATPase-ZL* | 80-100% | AP022238 |
| *A. baumannii* | KSK Sensitive | 2020 | India | Respiratory specimen | 374 | chr | 3841 | 872448.. 887625 | *ABCX-DUF262-ZL* | 76-99% | CP072305 |
| *A. baumannii* | DT0544C | 2017 | Tanzania | Clinical sample | 374 | chr | 3912 | 861905..877082 | *ABCX-DUF262-ZL* | 76-99% | CP053215 |
| *A. baumannii* | RCH52 | NA | NA | NA | 729 | chr | 4023 | 1350854..1365976 | *ABCX-DUF262-ZL* | 76-98% | CP085788 |
| *A. baumannii* | WKA02 | 2013 | South Korea | Sputum | 10 | chr | 3870 | 1288033..1293312 | *ABCX∆* | 97-98% | CP020598 |
| *A. baumannii* | PG20180064 | 2018 | NA | Mouse gut | NA | chr | 3843 | 2238215..2239054 | *LΔ* |  | CP043180 |
| *A. nosocomialis* | 2010S01-197 | 2010 | Taiwan | Homo sapiens/NA | 1603 | p2010S01-197-2 | 92 | 20894..27644 | *A∆BC∆X∆* | 99% | CP033563 |
| *A. nosocomialis* | 6411 | NA | NA | NA | 322 | p6411-89.111kb | 89 | 72590..87151 | *ABCX-HP-ZL* | 77-99% | CP010369 |
| *A. seifertii* | AS23 | 2010/2017 | Taiwan | Blood | 549 | pAS23-2 | 126 | 58877..73431 | *A∆BC∆X∆-HP-Z∆L∆* | 99% | CP061674 |
| *A. seifertii* | AS61 | 2010/2017 | Taiwan | Blood | 544 | pAS61-1 | 93 | 38440..54314 | *ABC∆X∆-HP, HP- Z∆-*ISAba26*-Z∆L* | 98-99 | CP061590 |
| *A. seifertii* | AS6 | 2010/2017 | Taiwan | Blood | NA | pAS6-1 | 123 | 113265..116157s | *CΔ* |  | CP061692 |
| *A. pittii* | DUT-2 | 2015 | China | Marine sediments | 640 | p1DUT-2^g^ | 141 | 108029..122531 | *ABCX-HP-ZL∆* | 95-100% | CP014652 |
| *A. pittii*. | Ac-14 | 2016 | China | Fruit tree rhizosphere soil | 640 | pAc-14_1^g^ | 117 | 100984..115487 | *ABCX-HP-ZL* | 95-100% | CP063770 |
| *A. pittii* | AB17H194 | 2017 | China | Wound secretion | 795 | pAB17H194-2 | 76 | 54184..69541 | *ABCX∆-HP-ZL* | 97-99% | CP040913 |
| *A. pittii* | IHIT24944 | 2014 | Germany | Canis lupus familiaris/ Nose | 93 | pAP24944-OXA-58 | 53 | 36368.. 51518 | *ABCX-DUF262-ZL* | 73-99% | KY888886 |
| *A. pittii* | WCHAP005069 | NA | China | Homo sapiens/NA | 64 | p1_005069 | 91 | 48597.. 63158 | *ABCX-HP-ZL* | 77-99% | CP026087 |
| *A. pittii* | CEP14 | 2018 | Czech Republic | Groundwater from chloroethene-contaminated soil at chemical factory | 93 | pCEP14_01 | 95 | 26566..41127 | *ABCX-HP-ZL* | 77-99% | CP084922 |
| *A. pittii* | FDAARGOS 1396 | NA | Germany | NA | 744 | p1FDAARGOS_1396^g^ | 94 | 52672..67233 | *ABCX-HP-ZL* | 77-99% | CP077304 |
| *A. pittii* | FDAARGOS_1215 | NA | Germany | NA | 248 | p2FDAARGOS_1215^g^ | 94 | 62732..77293 | *ABCX-HP-ZL* | 77-99% | CP069506 |
| *A. pittii* | FDAARGOS_1214 | NA | Germany | NA | 64 | p3FDAARGOS_1214^g^ | 94 | 36159..50720 | *ABCX-HP-ZL* | 77-99% | CP069540 |
| *A. pittii* | MS32 | NA | Taiwan | NA | NA | pMS32-1 | 94 | 53854..68416 | *ABCX∆-HP-ZL* | 94-99% | KJ616405 |
| *A. pittii* | 2012N21-164 | 2012 | Taiwan | Homo sapiens/NA | 248 | p2012N21-164-1 | 97 | 33080..47635 | *A∆B∆C∆X∆-HP-Z∆L∆* |  | CP033536 |
| *A. pittii* | 2014S07-126 | 2014 | Taiwan | Homo sapiens/NA | 64 | p2014S07-126-2 | 96 | 56548.. 71099 | *A∆B∆C∆X∆-HP∆-Z∆L∆* |  | CP033532 |
| *A. pittii* | AP43 | 2018 | China | Urine | 64 | pAP43-2 | 92 | Join(1.. 2583, 80298.. 92276) | *ABCX-HP-Z∆L∆* | 77-99% | CP043054 |
| *A. pittii* | WCHAP100020 | 2015 | China | Homo sapiens/NA | 63 | p1_100020 | 77 | 5894.. 15871 | *ABCX∆Z∆L* | 98-99% | CP027251 |
| *A. pittii* | C54 | 2014 | Australia | Clinical sample | 63 | pC54_002 | 76 | 13578.. 17407 | *Z∆L∆* |  | CP042366 |
| *A. pittii* | AB17H194 | 2017 | China | Wound secretion | 795 | pAB17H194-1 | 88 | 8668..9747 | *L∆* |  | CP040912 |
| *A. pittii* | HUMV-6483 | 2008 | Spain | Urine | 214 | p11 | 112 | 59587..60030 | *A∆* |  | CP021429 |
| *A. pittii* | WP2-W18-ESBL-11 | 2018 | Japan | Wastewater treatment plant effluent | 1606 | pWP2-W18-ESBL-11_1 | 87 | 58779..59776 | *A∆L∆* |  | AP021937 |
| *A. pittii* | JXA13 | 2019 | China | Dog | 795 | Chr | 3972 | 883685.. 898801 | *ABCX-DUF262-ZL* | 75-98% | CP054137 |
| *A. johnsonii* | XBB1 | NA | NA | NA | NA | pXBB1-8 | 117 | 19999..35094 | *ABCX-DUF262-ZL* | 82-99% | CP010358 |
| *A. johnsonii* | FDAARGOS_1094 | NA | Germany | NA | NA | p3FDAARGOS_1094^g^ | 82 | 43538.. 56765 | *ABCXZL* | 74-97% | CP068188 |
| *A. schindleri* | HZE23-1 | 2018 | China | Stool of goose | NA | pHZE23-1-1 | 138 | 68355.. 81585 | *ABCXZL* | 82-100% | CP044464 |
| *A. schindleri* | HZE33-1 | 2018 | China | Stool of goose | NA | pHZE33-1-1 | 132 | Join(1..2796, 121871..132305) | *ABCXZ∆L* | 82-100% | CP044475 |
| *A. schindleri* | HZE30-1 | 2018 | China | Stool of goose | NA | pHZE30-1-1 | 110 | 41045..52559 | *ABCXZL∆* | 79-99% | CP044484 |
| *A. schindleri* | H3 | 2012 | China | The condensate water of the Shenzhou-9 spacecraft | NA | p1H3^g^ | 223 | Join(1.. 2366, 212717.. 223569) | *ABCXZ∆L∆* | 76-97% | CP030755 |
| *A. schindleri* | SGAir0122 | 2014 | Singapore | Air | NA | pSGAir0122 | 156 | 5630.. 9182 | *Z∆L∆* |  | CP025619 |
| *A. indicus* | MMS9-2 | 2018 | China | Soil | NA | pMMS9-2-1 | 121 | 62097.. 75327 | *ABCXZL* | 82-100% | CP044451 |
| *A. indicus* | HY20 | 2018 | China | Feces | NA | pAI01 | 116 | 32297..45527 | *ABCXZL* | 82-100% | CP044019 |
| *A. indicus* | FS42-2 | 2017 | China | Stool of duck | NA | pFS42-2-1 | 140 | 13294.. 26536 | *AΔBCΔXΔZΔLΔ* |  | CP046596 |
| *A. indicus* | C15_T | 2015 | China | Feces of pig | NA | pC15-2 | 68 | 20089..33283 | *ABCXZL* | 80-100% | CP048656 |
| *A. indicus* | B18 | 2016 | China | Stool of pigeon | NA | pB18-2 | 136 | 93770..108335 | *ABCX-HP-ZL* | 80-99% | CP044457 |
| *A. indicus* | GXNN15X4 | 2019 | China | Feces of porcine | NA | pGXNN15 | 87 | 71736..84942 | *ABCXZL* | 78-99% | CP071318 |
| *A. indicus* | XG03 | 2017 | China | Feces of cow | NA | pXG-160kb | 160 | 2970.. 16176 | *ABCX-DUF262-ZL* | 80-99% | CP045124 |
| *A. indicus* | CMG3-2 | 2017 | China | Stool of goose | NA | pCMG3-2-1 | 120 | 90780..104887 | *ABCXZL* | 79-99% | CP044446 |
| *A. indicus* | XG01 | 2017 | China | Cow feces | NA | pXG01-X3 | 103 | Join(1.. 4691, 95094.. 103586) | *ABCXZL* | 81-99% | CP045136 |
| *A. indicus* | TQ18 | 2017 | China | Cow feces | NA | p18TQ-X3 | 93 | 91266..92345 | *LΔ* |  | CP045132 |
| *A. indicus* | LYS68A | 2019 | China | Feces of guinea fowl | NA | chr | 3212 | 1325209.. 1344016 | *ABCX-RecQ, DprA,, DUF262-ZL* | 82-99% | CP070997 |
| *A. indicus* | GXNN62X4 | 2019 | China | Feces of porcine | NA | chr | 3013 | 2886343.. 2899573 | *ABCXZL* | 82-99% | CP071319 |
| *A. indicus* | 94-2 | 2018 | China | Cow | NA | chr | 3124 | 2270108.. 2285222 | *ABCX-DUF262-ZL* | 75-99% | CP041291 |
| *A*. spp. | 10FS3-1 | 2015 | China | Feces of pig | NA | p10FS3-1-2 | 75 | 21123..34368 | *ABCXZL* | 72-99% | CP039145 |
|  |  |  |  |  |  | p10FS3-1-3 | 73 | 58953..60050 | *L∆* |  | CP039146 |
|  |  |  |  |  |  | p10FS3-1-4 | 54 | 9777..24883 | *ABCX-AAA family ATPase-ZL* | 82-100% | CP039147 |
| *A*. spp. | WCHA45 | 2015 | China | Sewage | NA | pNDM1_010045 | 190 | 131642.. 144809 | *ABCXZL* | 79-100% | CP028560 |
| *.* |  |  |  |  |  | pNDM1_010055 | 71 | 25239..40300 | *ABCX-AAA family ATPase, HP-ZL* | 78-99% | CP032284 |
| *A.* spp. | NEB 394 | 1986 | USA | Soil | NA | pBspH3 | 85 | 39398..52627 | *ABCXZL* | 74-100% | CP055285 |
| *A.* spp. | YH12138_T | 2017 | China | Pig/ Feces | NA | pYH12138-2 | 105 | 78496..91713 | *ABCXZL* | 78-99% | CP048672 |
| *A.* spp. | SH19PTT10 | NA | China | Pig/NA | NA | pYUSHP10-1 | 174 | 116266...129501 | *ABCXZL* | 77-99% | MT107270 |
| *A.* spp. | ACNIH2 | 2015 | USA | NA | NA | pACI-55cf | 118 | 62307..77400 | *ABCXZL* | 76-99% | CP026415 |
| *A.* spp. | Tol5 | NA | NA | NA | NA | chr | 4681 | 2686621..2701738 | *ABCX-DUF262-ZL* | 81-100% | AP024708 |
|  |  |  |  |  |  | pTol5 | 117 | 75365..88556 | *ABCXZL* | 79-99% | AP024709 |
| *A. radioresistens* | DSM 6976 = NBRC 102413 = CIP 103788 | NA | NA | Cotton sterilized by gamma rays | NA | pARA2 | 77 | 42130.. 55360 | *ABCXZL* | 78-100% | AP019742 |
| *A. radioresistens* | DD78 | 2008 | Chile | Soil | NA | pAR1 | 88 | Join(1..11696,85161.. 88584) | *ABCX-DUF262-Z∆L* | 75-99% | CP038023 |
| *A. radioresistens* | LH6 | 2017 | USA | poultry manure | NA | chr | 3089 | 2286167..2305005 | *ABCX-RecQ, DprA, DUF262-ZL* | 73-99% | CP030031 |
| *A. haemolyticus* | w12 | 2019 | China | Water | NA | p4 | 302 | 283159.. 296381 | *ABCXZ∆L∆* | 82-99% | CP085288 |
| *A. haemolyticus* | XH900 | 2016 | China | Blood | NA | pXH901 | 54 | 28572.. 41760 | *ABCXZL* | 75-99% | CP018261 |
| *A. haemolyticus* | AN43 | 2015 | Mexico | Peritoneal dialysis liquid | NA | pAhaemAN43b | 83 | 19686..34195 | *ABC-OprD-X∆-*IS*3-X∆ZL* | 96-100% | CP031977 |
| *A. haemolyticus* | AN59 | 2016 | Mexico | Peritoneal dialysis liquid | NA | pAhaemAN59c | 82 | 49365.. 62589 | *ABCXZL* | 75-100% | CP031973 |
| *A. haemolyticus* | 2126ch | 2011 | Mexico | Ascites liquid | NA | pAhaem2126chf | 70 | 35078.. 49631 | *ABCX-HP-ZL* | 71-100% | CP031992 |
| *A. haemolyticus* | TJS01 | 2012 | China | Homosapiens/NA | NA | pAHTJS1 | 56 | 34948.. 49501 | *ABCX-HP-ZL* | 71-100% | CP018872 |
| *A. lwofii* | ED9-5a | NA | Russia | Permafrost | NA | pALWED3.1 | 138 | 90651.. 103863 | *ABCXZL* | 74-99% | KX528687 |
| *A. lwofii* | EK30A | NA | Russia | Permafrost, Kolyma lowland | NA | pALWEK1.1 | 209 | 181225.. 195434 | *ABC∆-*IS*982- C∆XZL* | 74-99% | KX528688 |
| *A. lwofii* | FDAARGOS_551 | NA | USA | Clinical isolate | NA | p1FDAARGOS_551^g^ | 208 | Join(1..18331, 201236..208308) | *ABC∆-*IS*4*∆, IS*5*∆, *DUF4882Δ, type 1 fimbrial protein, fimbrial biogenesis outer membrane usher protein, fimbria/pilus periplasmic chaperone, type 1 fimbrial protein,* ISAba31, ISAba1∆-*C∆XZL∆-*IS*3*-*L∆* | 74-99% | CP054821 |
| *A. pseudolwoffii* | XMC5X702 | 2020 | China | A feces sample of chicken origin | NA | pXMC5X702-tetX-145k | 145 | 39513.. 52731 | *ABCXZL* | 78-100% | CP084302 |
|  |  |  |  |  |  | pXMC5X702-195k | 195 | Join(1..12035,192372.. 195429) | *ABC∆X-DUF262-ZL* | 80-98% | CP084301 |
| *A. piscicola* | YH12207_T | 2017 | China | Pig/Feces | NA | pYH12207-2 | 148 | 39408.. 52640 | *ABCXZL* | 77-99% | CP048661 |
| *A. wuhouensis* | WCHAW010062 | 2015 | China | Sewage | NA | pOXA58_010062 | 59 | 34704.. 47958 | *ABCXZL* | 74-100% | CP033131 |
| *A. variabilis* | FDAARGOS_1487 | NA | Germany | NA | NA | chr | 3211 | 953370..968571 | *ABCX-DUF262-ZL* | 69-99% | CP083658 |
|  |  |  |  |  |  | p2FDAARGOS_1487 ^g^ | 89 | 19013.. 20237 | *AB* | 98-99% | CP083660 |
| *A. variabilis* | XM9F202-2 | 2020 | China | A feces sample of chicken origin | NA | pXM9F202-2-186k | 186 | 31423.. 44666  67342..68745 | *ABCXZL-*IS*3*∆-*22315bp-L∆* | 77-99% | CP060812 |
| *A. bereziniae* | GD0320 | 2016 | China | Sputum | NA | pGD0320 | 122 | 5165..8179 | *ABC∆* | 97-100% | CP066122 |
| *A. bereziniae* | HPC229 | 2019 | Argentina | Blood | NA | pAbe229-114 | 114 | 65064.. 78260 | *ABCXZL* | 78-99% | CM012183 |
| *A. venetianus* | VE-C3 | 1997 | Italy | Seawater | NA | pAV3 | 186 | 76226.. 89447 | *ABCXZL* | 78-100% | NZ_ALIG01000010 |

^a^ The host is only indicated in the source column if the clinical sample is not obtained from the human host or its exact origin is unknown.

^b^ IP for Pasteur Institut MLST scheme.

^c^ If the BREX module is present in the chromosome, chr is mentioned. If it is present in the plasmid, the name of the plasmid is mentioned instead.

^d^ If an insertion sequence or an intruding gene is inserted, the name of the insertion sequence and the protein encoded by the gene is mentioned between dashes. If many genes or insertion sequences are listed between dashes, they are separated between commas. DUF262 corresponds to DUF262 domain-containing HNH endonuclease family protein-encoding gene, DprA to DNA-processing protein DprA encoding gene, RecQ to RecQ family ATP-dependent DNA helicase, HP to hypothetical protein-encoding gene.

^e^ compared to BREX proteins encoded by pCl107. Only complete proteins are compared.

^f^  NA for not available.

^g^ named here by preceding the strain name by the letter p.

^h^ Δ indicates incomplete, interrupted or frameshifted genes.
