# Supplementary material for: Analysis of pCl107 a large plasmid carried by an ST25 Acinetobacter baumannii strain reveals a complex evolutionary history and links to multiple antibiotic resistance and metabolic pathways
Source: FEMS Microbes. 2022 Nov 18;3:xtac027. doi: 10.1093/femsmc/xtac027 (PMC10117892; doi:10.1093/femsmc/xtac027)
Supplement: xtac027_Supplemental_Files [file xtac027_supplemental_files.zip › Table_S4_Supplementary_Data.docx]

**Table S4.** Properties of *Acinetobacter baumannii* strains with or without plasmids containing *hpxO*

| **Strain** | **Plasmid** | **Size** | **Year** | **Country** | **Source** | ***hpxO*** | **ST^IP a^** | **Uric acid module** | **aa identity range^b^** | **Genomic position** | **GenBank acc. no** |
| --- | --- | --- | --- | --- | --- | --- | --- | --- | --- | --- | --- |
| VB35179 | p1VB35179^c^ | 236 | 2018 | India | Blood | + | 1512 | *puuE∆^d^, uao, hiuH∆, uacT,* (ISAha2)^e^, *allA, alc, hpxO* | 100% | 103216..110222 | CP040054 |
| Ab-C102 | pAb-C102_2 | 67 | 2016 | Ghana | Blood | + | 1472 | *tsx, uacT,* (IS*3*∆, IS*982*∆), *hpxO∆* [2X]^f^ | 40% | 15954.. 20140  49503.. 53688 | CP051864 |
| PM194229 | pPM194229_1 | 226 | 2019 | India | BAL | + | 10 | *puuE, uao, hiuH, uacT,* (ISAha2), *allA, alc, hpxO∆* | 100% | 57846.. 64464 | CP050433 |
| A1429 | pA1429c | 205 | 2010 | China | Secretion | + | 108 | *puuE, uao, hiuH, uacT,* (ISAha2), *allA, alc, hpxO* | 100% | 133972..141409 | CP046899 |
| A297 RUH875 | pA297-3 | 200 | 1984 | Netherlands | NA^g^ | + | 1 | *puuE, uao, hiuH, uacT,* (ISAha2), *allA, alc, hpxO* | 100% | 108716..116153 | KU744946 |
| Cl107 | pCl107 | 198 | 2012 | Lebanon | Urine | - | 25 | *puuE, uao, hiuH, uacT* | - | 105067..108390 | CP098522 |
| D46 | pD46-4 | 207 | 2010 | Australia | Mid stream urine | - | 25 | *puuE, uao, hiuH, uacT* | 100% | 119343..122666 | MF399199 |
| MC75 | pMC75.1 | 150 | 2016 | Bolivia | Ulcer | - | 15 | *puuE, uao, hiuH, uacT* | 100% | 52579..55902 | MK531540 |
| UPAB1 | pAB5 | 100 | 2016 | Argentina | Urine | - | 25 | *puuE, uao, hiuH, uacT* | 100% | 95115..98435 | CP032216 |
| Acb-45063 | pAb45063_b | 183 | NA | NA | NA | - | NA | *puuE, uao, hiuH, uacT* | 100% | 128762..132085 | MK323043 |
| MC1 | pMC1.1 | 184 | 2015 | Bolivia | Catheter | - | 991 | *puuE, uao∆, hiuH, uacT∆* | 100% | 80185..83509 | MK531536 |

^a^ IP for Pasteur Institute MLST scheme.

^b^ compared to uric acid proteins (PuuE-Uao-HiuH-UacT) encoded by pCl107. Only complete proteins are compared.

^c^ named here by preceding the strain name by the letter p.

^d^ Δ indicates incomplete, interrupted or frameshifted genes.

^e^ If an insertion sequence is inserted, its name is mentioned between parenthesis.

^f^  The region is repeated twice.

^g^  NA for not available.
