# Supplementary material for: Analysis of pCl107 a large plasmid carried by an ST25 Acinetobacter baumannii strain reveals a complex evolutionary history and links to multiple antibiotic resistance and metabolic pathways
Source: FEMS Microbes. 2022 Nov 18;3:xtac027. doi: 10.1093/femsmc/xtac027 (PMC10117892; doi:10.1093/femsmc/xtac027)
Supplement: xtac027_Supplemental_Files [file xtac027_supplemental_files.zip › Table_S5_Supplementary_Data.docx]

**Table S5.** Distribution of uric acid metabolic modules in *Acinetobacter* spp.

| **Species names** | **Strain** | **Year** | **Country** | **Source** | **Genomic context^a^** | **Size (kb)** | **amino acid identity range^b^** | **Genomic position** | **Uric acid modules** | **GenBank acc. no.** |
| --- | --- | --- | --- | --- | --- | --- | --- | --- | --- | --- |
| *A. pittii* | DUT-2 | 2015 | China | Marine sediments | p1DUT-2^c^ | 141 | 100 | 48967..56404 | *puuE, uao, hiuH, uacT,* (ISAha2)^d^, *allA, alC, hpxO* | CP014652 |
| *A. pittii* | Ac-14 | 2016 | China | Fruit tree rhizosphere soil | p1Ac-14^c^ | 117 | 100 | 33402..36725 | *puuE, uao, hiuH, uacT* | CP063770 |
| *A. lowfii* | AL_065 | 2016 | Pakistan | Bedside rail in hospital intensive care unit | chr | 3308 | 100 | 1276314..1283580 | *puuE, uao, hiuH, uacT, tsxΔ^e^, allA, alC, hpxO* | CP078045 |
| *A. lowfii* | FDAARGOS 1394 | NA^f^ | Germany | NA | chr | 3226 | 100 | 2856780..2864046 | *puuE, uao, hiuH, uacT, tsxΔ, allA, alC, hpxO* | CP077369 |
| *A. lowfii* | FDAARGOS_551 | NA | USA | Clinical isolate | chr | 3307 | 99-100 | 2770194..2778857 | *puuE, uao, hiuH, uacT, tsx,* (ISAlw10-like), *allA, alC, hpxO* | CP054822 |
| *A. lowfii* | FDAARGOS_552 | NA | USA | Clinical isolate | chr | 3247 | 99-100 | 541903.. 550566 | *puuE, uao, hiuH, uacT, tsx,* (ISAlw10-like), *allA, alC, hpxO* | CP046296 |
| *A. lowfii* | EK30A | NA | Russia | Permafrost, Kolyma lowland | chr | 3187 | 99-100 | 2011590..2018854 | *puuE, uao, hiuH, uacT, tsx, allA, alC, hpxO* | CP080636 |
| *A. lowfii* | H7 | 2016 | China | Chickens | chr | 3040 | 72-86  100 | 807535..814631  337075..344341 | *puuE, uao, hiuH, uacTΔ, tsx, allA, alC, hpxO*  *puuE, uao, hiuH, uacT, tsxΔ, allA, alC, hpxO* | CP072549 |
| *A. lowfii* | FDAARGOS_557 | NA | USA | Clinical isolate | chr | 3513 | 73-87 | 1982193..1989288 | *puuE, uao, hiuH, uacT, tsx, allA, alC, hpxO* | CP054803 |
| *A. lowfii* | VS15 | NA | Russia | Permafrost, Kolyma lowland | chr | 3260 | 73-88 | 3080402..3087497 | *puuE, uao, hiuH, uacT, tsx, allA, alC, hpxO* | CP080576 |
| *A. lowfii* | ZS207 | 2012 | Poland | Microbial mats from Zloty Stok gold mine | chr | 3259 | 76-87 | 1699307..1706402 | *puuE, uao, hiuH, uacT, tsx, allA, alC, hpxO* | CP019143 |
| *A. lowfii* | FDAARGOS 1393 | NA | Germany | NA | chr | 3166 | 75-87 | 3040243..3047339 | *puuE, uao, hiuH, uacT, tsx, allA, alC, hpxO* | CP077336 |
| *A. variabilis* | FDAARGOS_1487 | NA | Germany | NA | chr | 3211 | 72-89 | 489459..496554 | *puuE, uao, hiuH, uacT, tsx, allA, alC, hpxO* | CP083658 |
| *A. variabilis* | RYU24 | 2012 | Japan | Human fecal | chr | 3198 | 71-89 | 1738638..1745733 | *puuE, uao, hiuH, uacT, tsx, allA, alC, hpxO* | AP024524 |
| *A. variabilis* | AV_175 | 2016 | Pakistan | Alcohol foam dispenser in hospital intensive care unit | chr | 3252 | 73-89 | 1670719..1677810 | *puuE, uao, hiuH, uacT, tsx, allA, alC, hpxO* | CP078027 |
| *A. schindleri* | HZE30-1 | 2018 | China | Goose/Stool | chr | 3097 | 63-84 | 2978986.. 2987054 | *puuE, uao, hiuH, uacT, tsx,* (GNAT family N-acetyltransferase), *hpxO, alC, allA* | CP044483 |
| *A. schindleri* | SGAir0122 | 2014 | Singapore | Air | chr | 3088 | 63-84 | 2970056..2978124 | *puuE, uao, hiuH, uacT, tsx,* (GNAT family N-acetyltransferase), *hpxO, alC, allA* | CP025618 |
| *A. schindleri* | HZE23-1 | 2018 | China | Goose/Stool | chr | 3135 | 62-85 | 3014344..3022399 | *puuE, uao, hiuH, uacT, tsx,* (GNAT family N-acetyltransferase), *hpxO, alC, allA* | CP044463 |
| *A. schindleri* | HZE33-1 | 2018 | China | Goose/Stool | chr | 3131 | 63-84 | 1457970..1466044 | *puuE, uao, hiuH, uacT, tsx,* (GNAT family N-acetyltransferase), *hpxO, alC, allA* | CP044474 |
| *A. schindleri* | H3 | 2012 | China | The condensate water of the Shenzhou-9 spacecraft | chr | 2973 | 62-84 | 134960..143032 | *puuE, uao, hiuH, uacT, tsx,* (GNAT family N-acetyltransferase), *hpxO, alC, allA* | CP030754 |
| *A. schindleri* | ACE | 2013 | Mexico | NA | chr | 3001 | 63-84 | 108059.. 116134 | *puuE, uao, hiuH, uacT, tsx,* (GNAT family N-acetyltransferase), *hpxO, alC, allA* | CP015615 |
| *A. tandoii* | SE63 | 2013 | China | Mangrove wetland ecosystem | chr | 3543 | 45-78 | 726215..734137  3495403.. 3497610 | *uao, hiuH, uacT,* (HP)*, hpxO,* (FadR family transcriptional regulator), *alC, allA*  *puuE, tsx* | CP041365 |
| *A. cumulans* | WCHAc060092 | 2018 | China | Sewage | chr | 3427 | 72-98 | 1748906..1755343 | *puuE, uao, hiuH, uacT, allA, alC, hpxO* | CP035934 |
| *A. junii* | ZM06 | 2018 | China | Soil | chr | 3640 | 45-77 | 96338..98551  2697024..2704947 | *puuE, tsx*  *uao, hiuH, uacT,* (HP)*, hpxO,* (FadR family transcriptional regulator), *alC, allA* | CP077415 |
| *A. johnsonii* | FDAARGOS_1092 | NA | Germany | NA | p1FDAARGOS_1092^c^ | 183 | 100 | 4738..12002 | *puuE, uao, hiuH, uacT, tsx, allA, alC, hpxO* | CP068202 |
| *A. spp.* | ACNIH1 | 2014 | USA | NA | chr | 3307 | 99-100  72-89 | 915065..922329  1389487..1396579 | *puuE, uao, hiuH, uacT, tsx, allA, alC, hpxO*  *puuE, uao, hiuH, uacT, tsx, allA, alC, hpxO* | CP026420 |
|  |  |  |  |  | pACI-3569 | 127 | 100 | 59142..66406 | *puuE, uao, hiuH, uacT, tsx, allA, alC, hpxO* | CP026416 |
| *A*. spp. | NEB149 | NA | NA | NA | chr | 3218 | 73-87 | 1559164..1566257 | *puuE, uao, hiuH, uacT, tsx, allA, alCΔ, hpxO* | CP051208 |
| *A.* spp. | FDAARGOS_724 | NA | NA | Clinical isolate | chr | 3235 | 74-90 | 2204957..2212049 | *puuE, uao, hiuH, uacT, tsx, allA, alC, hpxO* | CP054908 |
| *A.* spp. | WY4 | 2016 | Antarctica | Euphausia superba | chr | 3123 | 58-88 | 418323..425420 | *puuE, uao, hiuHΔ, uacT, tsx, allA, alC, hpxO* | CP053947 |
| *A.* spp. | YH12138_T | 2017 | China | Pig/feces | chr | 3317 | 83-93 | 1713633..1720136 | *puuE, uao, hiuH, uacT, allA, alC, hpxO* | CP048670 |
| *A.* spp. | Tol5 | NA | NA | NA | pTol5 | 117 | 100 | 22038..32692 | *puuE,* (IS*4*Δ), *uao, hiuH, uacT, tsxΔ,* (HP, IS*1*, IS*629* transposase OrfB), *allA, alC, hpxO* | AP024709 |
| *A.* spp. | NCu2D-2 | 2014 | Germany | Tracheal swab from cat-captured mouse | pNCu2D-2 | 309 | 72-88 | 98769.. 110220 | *puuE,* (transposaseΔ, RNA-guided endonuclease TnpB family protein), *uao, hiuH, uacTΔ,* (IS*200*/IS*605*, RNA-guided endonuclease TnpB family protein, HP)*, tsx, allA, alC, hpxO* | CP015595 |

^a^ If the uric acid module is present in the chromosome, chr is mentioned. If it is present in the plasmid, the name of the plasmid is mentioned instead.

^b^ compared to uric acid proteins (PuuE-Uao-HiuH-UacT) encoded by pCl107. Only complete proteins are compared.

^c^ named here by preceding the strain name by the letter p.

^d^ If an insertion sequence or an intruding gene is inserted, the name of the insertion or the protein encoded by the gene is mentioned between parenthesis.

^e^ Δ indicates incomplete, interrupted or frameshifted genes.

^f^  NA for not available.
